# Treatment of COVID-19-associated ARDS with mesenchymal stromal cells: a multicenter randomized double-blind trial

**DOI:** 10.1186/s13054-022-03930-4

**Published:** 2022-02-21

**Authors:** Antoine Monsel, Caroline Hauw-Berlemont, Miryam Mebarki, Nicholas Heming, Julien Mayaux, Otriv Nguekap Tchoumba, Jean-Luc Diehl, Alexandre Demoule, Djillali Annane, Clémence Marois, Sophie Demeret, Emmanuel Weiss, Guillaume Voiriot, Muriel Fartoukh, Jean-Michel Constantin, Bruno Mégarbane, Gaëtan Plantefève, Stéphanie Malard-Castagnet, Sonia Burrel, Michelle Rosenzwajg, Nicolas Tchitchek, Hélène Boucher-Pillet, Guillaume Churlaud, Audrey Cras, Camille Maheux, Chloé Pezzana, Mamadou Hassimiou Diallo, Jacques Ropers, Philippe Menasché, Jérôme Larghero, APHP STROMA–CoV-2 Collaborative Research Group, Déborah Benchetrit, Harold Bonvallot, Fanny Charbonnier-Beaupel, Meriem Dhib-Charfi, Pierre Romain Delmotte, Assitan Kone, Marine Le Corre, Anne-Geneviève Marcelin, Carole Metz, Louis Puybasset, Joe-Elie Salem, Corinne Vezinet

**Affiliations:** 1 Multidisciplinary Intensive Care Unit, Department of Anesthesiology and Critical Care, La Pitié–Salpêtrière Hospital, Assistance Publique-Hôpitaux de Paris (APHP) Sorbonne University, Paris, France; 2 Sorbonne Université–INSERM UMRS_959, Immunology–Immunopathology–Immunotherapy (I3), 75013 Paris, France; 3 Biotherapy (CIC-BTi), Hôpital Pitié-Salpêtrière, APHP, 75651 Paris, France; 4 Intensive Care Unit, APHP-CUP, Hôpital Européen Georges-Pompidou, Université de Paris, 75015 Paris, France; 5 APHP, Hôpital Saint-Louis, Unité de Thérapie Cellulaire, Centre d’Investigation Clinique en Biothérapies CBT501, INSERM, Université de Paris, Paris, France; 6 FHU SEPSIS, Department of Intensive Care, Hôpital Raymond-Poincaré (APHP), Laboratory of Infection & Inflammation–INSERM U1173, Simone Veil School of Medicine, University Versailles Saint Quentin–University Paris Saclay, 92380 Garches, France; 7 APHP, Groupe Hospitalier Universitaire–Sorbonne Université, site Pitié–Salpêtrière, Service de Médecine Intensive et Réanimation (Département R3S), and Sorbonne Université, INSERM, UMRS1158 Neurophysiologie Respiratoire Expérimentale et Clinique, Paris, France; 8 Innovative Therapies in Hemostasis, INSERM, 75006 Paris, France, and Biosurgical Research Laboratory (Carpentier Foundation), APHP-CUP, Hôpital Européen Georges-Pompidou, Université de Paris, 75015 Paris, France; 9 Neurological Intensive Care Unit, Department of Neurology, La Pitié–Salpêtrière Hospital, APHP, Sorbonne University, Paris, France; 10 Groupe de Recherche Clinique en REanimation et Soins intensifs du Patient en Insuffisance Respiratoire aiguE (GRC-RESPIRE), Sorbonne Université, Paris, France; 11 Department of Anesthesiology and Critical Care, Beaujon Hospital, DMU PARABOL, APHP Nord, Paris, France; 12 Center for Research on Inflammation, INSERM and Université de Paris, Paris, France; 13 Service de Médecine Intensive Réanimation, Hôpital Tenon, APHP, Sorbonne Université, Paris, France; 14 Department of Medical and Toxicological Critical Care, Lariboisière Hospital, INSERM UMRS1144, University of Paris, Paris, France; 15 Service de Réanimation Polyvalente, Centre Hospitalier Victor Dupouy, 69, Rue du Lieutenant-Colonel Prud’hon, 95100 Argenteuil, France; 16 Immunology and HLA Laboratory, Hôpital Saint-Louis, Paris, France; 17 INSERM U1136, Institut Pierre-Louis d’Epidémiologie et de Santé Publique (iPLESP), APHP, Hôpital Pitié–Salpêtrière, Service de Virologie, Sorbonne Université, Paris, France; 18 APHP, Hôpital Saint-Louis, Centre MEARY de Thérapie Cellulaire et Génique, Paris, France; 19 INSERM UMR1140, Université de Paris, 75006 Paris, France; 20 INSERM, UMR S 970, Paris Centre de Recherche Cardiovasculaire (PARCC), Université de Paris, Paris, France; 21 Clinical Research Unit, Pitié–Salpêtrière University Hospital, APHP, Paris, France; 22 Department of Cardiovascular Surgery, Hôpital Européen Georges-Pompidou, Paris, France; 23 Multidisciplinary Intensive Care Unit, Department of Anesthesiology–Critical Care and Perioperative Medicine, Hôpital de la Pitié–Salpêtrière, 47–83, boulevard de l’Hôpital, 75651 Paris Cedex 13, France; 24 Internal Use Pharmacy Department, REQPHARM Unit, La Pitié–Salpêtrière Hospital, Assistance Publique-Hôpitaux de Paris (APHP) Sorbonne University, Paris, France; 25 Clinical Investigation Center (CIC) at La Pitié–Salpêtrière Hospital, Assistance Publique-Hôpitaux de Paris (APHP), Sorbonne University, Paris, France

**Keywords:** Severe acute respiratory syndrome coronavirus-2, Acute respiratory distress syndrome, Umbilical cord-derived mesenchymal stromal cells, Good-manufacturing practice

## Abstract

**Background:**

Severe acute respiratory syndrome coronavirus-2 (SARS–CoV-2)-induced acute respiratory distress syndrome (ARDS) causes high mortality. Umbilical cord-derived mesenchymal stromal cells (UC-MSCs) have potentially relevant immune-modulatory properties, whose place in ARDS treatment is not established. This phase 2b trial was undertaken to assess the efficacy of UC-MSCs in patients with SARS–CoV-2-induced ARDS.

**Methods:**

This multicentre, double-blind, randomized, placebo-controlled trial (STROMA–CoV-2) recruited adults (≥ 18 years) with SARS–CoV-2-induced early (< 96 h) mild-to-severe ARDS in 10 French centres. Patients were randomly assigned to receive three intravenous infusions of 10^6^ UC-MSCs/kg or placebo (0.9% NaCl) over 5 days after recruitment. For the modified intention-to-treat population, the primary endpoint was the partial pressure of oxygen to fractional inspired oxygen (PaO_2_/FiO_2_)-ratio change between baseline (day (D) 0) and D7.

**Results:**

Among the 107 patients screened for eligibility from April 6, 2020, to October 29, 2020, 45 were enrolled, randomized and analyzed. PaO_2_/FiO_2_ changes between D0 and D7 did not differ significantly between the UC-MSCs and placebo groups (medians [IQR] 54.3 [− 15.5 to 93.3] vs 25.3 [− 33.3 to 104.6], respectively; ANCOVA estimated treatment effect 7.4, 95% CI − 44.7 to 59.7; *P* = 0.77). Six (28.6%) of the 21 UC-MSCs recipients and six of 24 (25%) placebo-group patients experienced serious adverse events, none of which were related to UC-MSCs treatment.

**Conclusions:**

D0-to-D7 PaO_2_/FiO_2_ changes for intravenous UC-MSCs-versus placebo-treated adults with SARS–CoV-2-induced ARDS did not differ significantly. Repeated UC-MSCs infusions were not associated with any serious adverse events during treatment or thereafter (until D28). Larger trials enrolling patients earlier during the course of their ARDS are needed to further assess UC-MSCs efficacy in this context.

*Trial registration*: NCT04333368. Registered 01 April 2020, https://clinicaltrials.gov/ct2/history/NCT04333368.

**Supplementary Information:**

The online version contains supplementary material available at 10.1186/s13054-022-03930-4.

## Background

Severe acute respiratory syndrome coronavirus-2 (SARS–CoV-2) causes coronavirus disease-2019 (COVID-19) and is frequently fatal, with mortality of its most severe forms averaging 30–40% and necessitating intensive care unit (ICU) admission [1]. A major root cause of those deaths is uncontrolled immune-system dysregulation, leading to, among others, alveolocapillary membrane damage evolving into acute respiratory distress syndrome (ARDS), which requires mechanical ventilation in up to 90% of ICU patients [2]. One of the hallmarks of COVID-19-associated ARDS is a dysregulated immune response, characterized by a shift of immune cells and their secreted cytokines toward an inflammatory pattern [3]. So far, systemic corticosteroid administration has been shown to lower the 28-day mortality rate of critically-ill COVID-19 patients [3]. Likewise, interleukin (IL)-6-receptor blockers have been reported to improve outcomes (organ-support-free days and survival) [4]. However, the challenges of implementing the vaccination strategy and the emergence of new viral variants still contribute to the persistently high mortality of patients with SARS–CoV-2-induced ARDS [5]. Those findings justify the ongoing quest for new therapies, among which mesenchymal stromal cells (MSCs) are gaining increased interest.

MSCs have well-documented, anti-inflammatory and immune-modulatory properties [6], which have supported their use in treating diseases whose pathophysiologies harbor a major inflammatory component [7]. Specifically, their anti-apoptotic, anti-oxidative, and tissue-reparative properties, diminishing lung vascular and epithelial permeability to proteins, and enhancing clearance of alveolar oedema fluid, have been demonstrated [8]. Furthermore, their capacity to temporarily evade the immune system [9] allows an allogeneic use, which streamlines the logistics of their clinical implementation. In addition, the results of a recent systematic review and meta-analysis of 55 clinical trials demonstrated their satisfactory safety profile [10]. Importantly, MSCs require priming by inflammatory signals to activate their immunomodulatory functions [11], and some of those signals have been shown to be involved in the pathophysiology of ARDS. Furthermore, their predominant pulmonary lodging following intravenous infusion also argue in favour of administering them to patients with ARDS. As a matter of fact, MSCs have been administered intravenously in phase 1 and 2 clinical trials in more than 150 critically-ill ARDS patients with excellent results in terms of clinical tolerance and even some benefits on the modulation of inflammation biomarkers [12–14]. The combination of those features makes MSCs appealing candidates for treating SARS–CoV-2-induced pulmonary inflammation, as they might have a broader spectrum of action than drugs, which usually have a more limited number of targets. While MSCs can be harvested from various tissue sources, those from the umbilical cord (UC) Wharton’s jelly have distinct advantages over bone marrow- or fat tissue-derived MSCs: easy and non-invasive harvesting procedure, good clinical tolerance [15], excellent in vitro scalability and slower time to senescence. Perhaps of greater relevance in the specific context of COVID-19-induced pulmonary damage, UC-MSCs are credited with stronger angiogeneic [16, 17] and immunomodulatory properties [18, 19]. This is well-exemplified by the recent case report of a patient with COVID-19 respiratory failure, who received an intravenously infused of UC-MSCs, and whose in-depth immune profiling of peripheral blood and bronchoalveolar fluid lavage samples revealed: normalization of the circulating T-lymphocytes count, and reductions of inflammatory myeloid cells, serum levels of proinflammatory cytokines and lung-infiltrating inflammatory neutrophils, while circulating monocytes and low-density gradient neutrophils acquired immunosuppressive functions [20]. These UC-MSCs characteristics, possibly attributable to their more primitive origin compared with adult tissue-derived MSCs [18], might explain why those of cord origin were the most widely used according to a recent review of registered trials testing MSCs in COVID-19 patients [21]. In the specific context of SARS–CoV-2-induced severe ARDS, another reason for using UC-MSCs, is that they do not express the angiotensin-converting enzyme-2 receptor [22], unlike MSCs originating from other tissue types [23].

Studies testing UC-MSCs in COVID-19 patients published so far have consisted of anecdotal case reports [24, 25], small-sized non-randomized or open-label studies [22, 26–29] and only two recent, single-centre, double-blind, placebo-controlled trials [30, 31]. Overall, those studies’ results confirmed the excellent tolerance of intravenous MSC infusions and suggested improved clinical outcomes, although their interpretation is complicated by the broad heterogeneity of patients’ pre-treatment profiles, therapeutic doses and timing of treatment administration, cell tissue source, the different passage numbers at which cells were collected and the diversity of additional treatments, which generate strong background noise. We therefore designed this multicentre, double-blind, randomized, placebo-controlled STROMA–CoV-2 trial was designed to determine whether repeated intravenous infusions of UC-MSCs derived from Wharton’s jelly during the early stage of SARS–CoV-2-related ARDS could improve its resolution and impact circulating levels of biomarkers.

## Methods

A detailed description of the methods and the full clinical trial protocol are provided in the Additional file 1.

### Study design

The multicentre, double-blind, randomized, placebo-controlled STROMA–CoV-2 trial was designed to compare the intravenous infusion of UC-MSCs versus saline placebo as add-on therapy for the management of SARS–CoV-2-induced ARDS. The study was conducted in ten ICUs in eight French university hospitals. The National Review Board of Île-de-France III approved the trial (CNRIPH 20.03.26.39722) that was authorized by the French National Agency for Medicines and Health Products Safety (EudraCT 2020-001287-28). The trial is registered with ClinicalTrals.gov identifier NCT04333368. A Data-Safety-Monitoring Board reviewed serious adverse events and the results after 10, 20 and 40 patients had been enrolled.

### Patients

Eligible patients had Berlin criteria-defined ARDS (mild-to-severe) for < 96 h, reverse transcriptase-polymerase chain reaction (RT-PCR)-confirmed SARS–CoV-2 infection, and were receiving respiratory support (invasive or non-invasive mechanical ventilation, and/or high-flow nasal oxygenation, with positive end-expiratory pressure equivalent ≥ 5 cmH_2_O). Patients with pulmonary embolism, immunocompromised status, liver disease, chronic lung disease or cancer were excluded. The detailed list of exclusion criteria is given in Table 1. Written informed consent was obtained from patients or a legally designated representative.

**Table 1:** Inclusion and exclusion criteria

Inclusion criteria	Exclusion criteria
Age > 18 years Reverse transcriptase–polymerase chain reaction -confirmed SARS–CoV-2 infection Berlin criteria-defined acute respiratory distress syndrome for < 96 h Respiratory support (invasive or non-invasive mechanical ventilation, and/or high-flow nasal oxygenation) with positive end-expiratory pressure equivalent ≥ 5 cm H_2_O	Age < 18 years Acute respiratory distress syndrome present for > 96 h Pulmonary fibrosis Pulmonary hypertension (WHO classification class III or IV) Pulmonary embolism within the previous 3 months Extracorporeal membrane oxygenation or life support Immunocompromised status including use of immunosuppressive medications Pregnancy or breastfeeding Treatment for cancer in the past 2 years Underlying medical condition with life expectancy < 6 months Moderate-to-severe liver disease (Child–Pugh score > 12) Severe chronic lung disease with the use of home oxygen and/or partial arterial pressure of carbon dioxide > 50 mm Hg Patients not committed to full support (i.e., had do not resuscitate or limit life support orders) Participation in another trial of COVID-19 therapeutics

### Randomization and blinding

Subjects were initially randomized at a 1:2 ratio to receive either the total dose of 3 × 10^6^ UC-MSCs/kg in 150 mL of 0.9% NaCl/0.5% albumin or placebo (150 mL of 0.9% NaCl) over 5 days, which was modified by a protocol amendment to 1:1 when patients became scarce at the end of the first wave. Randomization was stratified according to age (≤ 70 vs > 70 years) and the inclusion-day (D0) Sequential Organ-Failure Assessment (SOFA) score (≤ 11 vs > 11). All healthcare providers and patients were unaware of treatment assignment; only cell-production–unit staff members were not blinded.

### Production of the UC-MSCs-based advanced therapy medicinal product

The study treatments (UC-MSCs or placebo) were prepared by the Cell Therapy Unit and the MEARY Cell and Gene Therapy Center, which are two adjacent buildings, located in the same hospital (Saint Louis Hospital, Paris, France). Briefly, the investigational advanced therapy medicinal product was a suspension of allogenic UC-MSCs, isolated from human UC Wharton’s jelly by enzymatic digestion or the explant method, and amplified in vitro. Quality controls (Additional file 1: Table S1, S2 and S3) included viability, identity, purity, functionality (clonogenicity, immunosuppressive effects) and safety (microbiological, endotoxin and mycoplasma assays; karyotype).

### Procedures

Each patient received three intravenous infusions of 10^6^ UC-MSCs/kg (maximum dose set at 80 × 10^6^ cells per infusion) or placebo on D1, D3 ± 1 and D5 ± 1. All patients were monitored for any changes of pre-specified respiratory or cardiovascular parameters (see Additional file 1) and were ventilated according to the modified ARDS Network lower tidal volume protocol. Management of ARDS, septic shock and other organ failures followed international guidelines [32, 33].

### Clinical and biological outcomes

The primary endpoint of the study was respiratory improvement assessed as the partial pressure of oxygen to fractional inspired oxygen (PaO_2_/FiO_2_)-ratio change between baseline (D0) and D7 post-randomization. Secondary and safety endpoints are provided in the Additional file 1. To assess UC-MSCs biological activity, biomarkers of endothelial, alveolar epithelial injury and inflammation were measured in plasma obtained on D0, D2, D4, D7, and D14, as exploratory endpoints. SARS–CoV-2 nucleocapsid antigenemia (N-antigenemia) and viral RNA levels were measured in plasma on D1 and D7. The level of donor-specific anti-human leucocyte antigen (HLA) antibodies (DSAs) directed against UC-MSCs was also measured on D0 and D14 to detect allo-immunization.

### Statistical analyses

Continuous data are expressed as mean ± standard deviation or median [interquartile range, IQR]. Categorical parameters are expressed as numbers (percentages).

The primary endpoint was the evolution of the PaO_2_/FiO_2_ ratio between D0 and D7. PaO2/FiO2 ratios of patients who died were imputed to 50, considered a minimum possible value. Given the lack of literature data available at the time, the last observation carried forward (LOCF) plus 10% imputation for the missing PaO_2_/FiO_2_ ratios of patients who improved and were discharged from the ICU seemed reasonable and consistent with their clinically observed recovery dynamics. A sensitivity analysis was computed using a LOCF approach. The mean difference between the two groups was compared using analysis of covariance (ANCOVA), adjusting for the D0 PaO_2_/FiO_2_ ratio and stratification factors. Wilcoxon rank-sum test was used to analyse robustness. Statistical analyses used a two-sided 5% threshold of significance.

The principal analysis was conducted according to modified intent-to-treat, including all randomized patients, except those who received no dose of the assigned treatment. A per-protocol analysis was also conducted on patients who had received the three planned doses of the assigned treatment.

The evolutions of the criteria of interest were analysed at different times. Simple comparisons used Student’s, Wilcoxon’s, χ^2^, or Fisher’s exact tests according to the type of data. The evolution of clinical criteria, especially respiratory, was subjected to longitudinal modeling. Random-effects models were used to take into account a subject’s repeated measurements. Biomarkers were log_10_-transformed before analysis. Left-censoring (values below the limit of quantification) was accounted for either by modeling or imputation using each biomarker’s half-value of the limit of quantification. In light of the exploratory nature of the study, no penalty for the multiplicity of comparisons was implemented, except for cytokines that were subjected to Benjamini–Hochberg correction.

The sample size was chosen pragmatically in consideration of the capacity to produce UC-MSCs during a period when the need was urgent. However, the simulation run, which was meant to be only illustrative, suggested that the study should be able to demonstrate a 50% PaO_2_/FiO_2_-ratio increase from D0 to D7, based on the first information available on the PaO_2_/FiO_2_-ratio distributions of COVID-19 patients in ICUs.

All analyses and calculations were computed using R software version 4.0.3, R Core Team (2020), R Foundation for Statistical Computing, Vienna, Austria.

## Results

### Patients

**Fig. 1:**
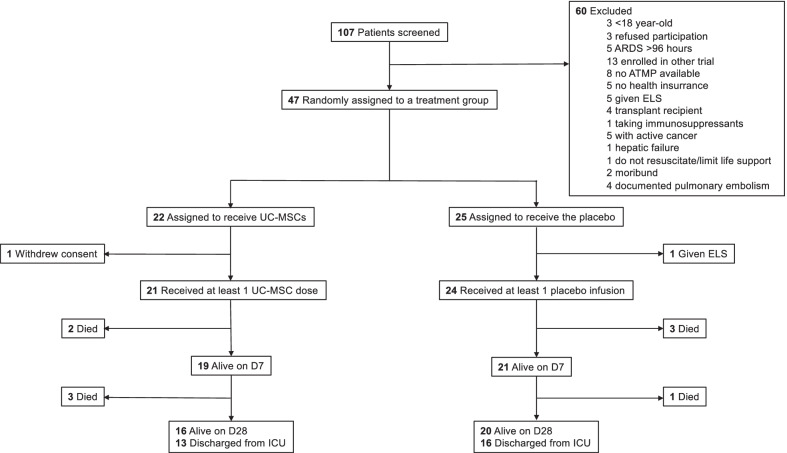
Flowchart of the trial. *ARDS* acute respiratory distress syndrome. *ATMP* advanced therapeutic medicinal product. *D* day. *ELS* extracorporeal life support. *ICU* intensive care unit. *UC-MSCs* umbilical cord-derived mesenchymal stromal cells

Patients were enrolled from April 6, 2020, to October 29, 2020, in the ICUs at ten study sites. Among the 107 patients screened for eligibility, 47 were randomly assigned to a treatment group and 45 received either UC-MSCs (*n* = 21) or placebo (*n* = 24; Fig. 1). Their demographic characteristics are reported in Table 2. ARDS was mild, moderate or severe, respectively, in 31.1%, 48.9% and 20% of the patients. While SOFA and lung injury scores, and PaO_2_/FiO_2_ ratio were similar for the two groups, more placebo-group patients were on invasive mechanical ventilation, receiving vasopressors and neuromuscular blocking agents. Fifteen (71.4%) and 19 (79.2%) patients in the UC-MSC and placebo groups, respectively, received corticosteroids during the first 7 days (Table 3). During the first 28 days, the two groups had similar respiratory characteristics (Additional file 1: Table S4).

**Table 2:** Patients’ baseline characteristics

	UC-MSC (*n* = 21)	Placebo (*n* = 24)	*p* value
Age, years	64 (10.4)	63.2 (11.4)	0.82
Male sex	17 (81%)	20 (83.3%)	1
Body mass index	28.6 (3.5)	28 (5.5)	0.68
Obesity	7 (33.3%)	6 (25%)	0.54
Sepsis-related Organ-Failure Assessment score	5.5 (2.7)	5.9 (2.7)	0.64
Mean arterial pressure, mm Hg	91.3 (18.3)	81.5 (16.9)	0.07
On vasopressors	5 (23.8%)	14 (58.3%)	0.02
Comorbidities			
Chronic obstructive pulmonary disease	0 (0%)	1/15 (6.7%)	1
Active smoking	0 (0%)	0 (0%)	0.24
Chronic heart failure	0 (0%)	0 (0%)	1
Atrial fibrillation	2/15 (13.3%)	0 (0%)	0.21
Hypertension	11/15 (73.3%)	10/15 (66.7%)	0.47
Coronary artery disease	2/15 (13.3%)	2/15 (13.3%)	1
Stroke	2/15 (13.3%)	1/15 (6.7%)	0.59
Immunodeficiency	0 (0%)	0 (0%)	1
Active neoplasia	0 (0%)	0 (0%)	1
Chronic corticosteroid intake	0 (0%)	0 (0%)	1
Immunomodulatory drugs	2/17 (11.8%)	0 (0%)	0.2
Respiratory characteristics			
Ventilatory support (NIV and/or HFNO)	10 (47.6%)	4 (16.7%)	0.02
Invasive mechanical ventilation	11 (52.4%)	20 (83.3%)	0.02
Tidal volume, mL/kg PBW	6.2 (0.7, *n* = 11)	6.3 (0.8, *n* = 20)	0.72
Plateau airway pressure, cm H_2_O	21.8 (4.2, *n* = 10)	24.8 (5.1, *n* = 17)	0.12
PEEP	10.8 (2.9, *n* = 11)	11.2 (3.2, *n* = 20)	0.72
Driving pressure	11.3 (4.3, *n* = 10)	13.2 (3.9, *n* = 17)	0.25
Compliance, mL/cm H_2_O	45.2 (27.8, *n* = 10)	35.2 (14.9, *n* = 17)	0.29
SpO_2,_ %	94.6 (3.4)	96.0 (3.0, *n* = 23)	0.16
PaO_2_/FiO_2,_ mm Hg	156.2 (68.2)	171.2 (72.9)	0.53
Lung injury score	3.0 (0.7)	2.8 (0.5)	0.61
PaCO_2_, mm Hg	40 (8.5)	43.2 (9.8)	0.17
pH	7.41 (0.1)	7.37 (0.1)	0.27
Neuromuscular blocking agents	6 (28.6%)	16 (66.7%)	0.01
Ventilation mode			
Volume control	11/11 (100%)	19/20 (95%)	1
Pressure control	0 (0%)	0 (0%)	1
Pressure support	0 (0%)	1/20 (5%)	1

Values are expressed as mean (standard deviation) or number (%). Information was available for all patients, unless indicated otherwise

*HFNO* high-flow nasal oxygen therapy, *NIV* non-invasive ventilation, *PaCO*_*2*_ partial pressure of arterial carbon dioxide, *PaO*_*2*_*/FiO*_*2*_ ratio of partial pressure of oxygen to fractional inspired oxygen, *PBW* predicted body weight, *PEEP* positive end-expiratory pressure, *SpO*_*2*_ peripheral capillary oxygen saturation, *UC-MSCs* umbilical cord-derived mesenchymal stromal cells

**Table 3:** Assigned treatment doses received and corticosteroid administration from day 0 to day 7

	UC-MSCs (*n* = 21)	Placebo (*n* = 24)
Number of doses received over 7 days	2.7 (0.6)	2.7 (0.5)
One	2 (9.5%)	1 (4.2%)
Two	2 (9.5%)	4 (16.7%)
Three	17 (81%)	19 (79.2%)
Corticosteroids administered for 7 days	15 (71.4%)	19 (79.2%)

Values are expressed as number (%)

*UC-MSCs* umbilical cord-derived mesenchymal stromal cells

### Treatment

During the 5-day treatment period, 81.0%, 9.5% and 9.5% of UC-MSC recipients received three, two and one cell infusions, respectively (Table 3); they received a mean of 0.9 ± 0.1 × 10^6^ UC-MSCs/kg per dose (range 0.6–1 × 10^6^ UC-MSCs/kg) (Additional file 1: Table S5). Two (4%) UC-MSC batches did not meet specifications because of insufficient cell counts (< 1.00 ± 0.1 × 10^6^/kg) (Additional file 1: Table S5). Cell viability was 78.4 ± 5.3% and consistent across all batches (≥ 70%) (Additional file 1: Tables S2 and S3). UC-MSCs expressed CD90 (99.2 ± 1.6%), CD73 (99.9 ± 0.1%), and CD105 (97.0 ± 1.9%), while CD45, CD34, CD11b, CD19, and HLA-DR (0.8 ± 0.7%) were below their defined positivity thresholds (2%). The colony-forming unit–fibroblast assay yielded a frequency of 1.8 ± 1.1% and all batches satisfied the > 1% specification. Mixed lymphocyte-reaction assays, run to confirm UC-MSC immunomodulatory properties, showed significant dose-dependent inhibition of T-cell proliferation, with a peak inhibitory rate of 86 ± 5% for a UC-MSC/peripheral blood mononuclear cell ratio of 1:1.

### Primary endpoint

Of the 21 and 24 patients randomized to the UC-MSC and placebo groups, respectively, 17 and 18 of them had D7 primary endpoint measurements available. The respective numbers of patients whose D7 PaO_2_/FiO_2_-ratio values had to be imputed were four (three discharged and one died) and six (five discharged and one died). The primary outcome measure (PaO_2_/FiO_2_-ratio change between D0 and D7 post-randomization) did not differ significantly between the UC-MSC and placebo groups (median imputed values: 54.3 [IQR − 15.5 to 93.3] vs 25.3 [IQR − 33.3 to 104.6], respectively; ANCOVA treatment-effect estimate 7.4, 95% CI − 44.7 to 59.7; *P* = 0.77, Table 4).

**Table 4:** Main clinical outcomes

Primary endpoint	UC-MSCs (*n* = 21)	Placebo (*n* = 24)	Estimate (95% CI)	*p* value
PaO_2_/FiO_2_-ratio change D0–D7(principal analysis)	54.3 [− 15.5; 93.3]	25.3 [− 33.3; 104.6]	7.4 (− 44.7; 59.7)	0.77
PaO_2_/FiO_2_-ratio change D0–D7(sensitivity analysis)	54.3 [− 15.5; 93.3]	25.3 [− 33.3; 83.1]	12.5 (− 33.8; 56.7)	0.59

Secondary endpoints	UC-MSCs (*n* = 21)	Placebo (*n* = 24)	Median difference (95% CI), HR or sub-HR	*p* value
PaO_2_/FiO_2_-ratio change D0–D14	11.0 [− 39; 72.7]	28.2 [− 1; 67.3]	1.69 (− 82.1; 90.7)	1
Ventilation-free days to D28, *n*	17.0 [0; 25.0]	12.0 [0; 19.7]	0.5 (− 3.0; 8.0)	0.61
Ventilation duration to D28, *n*	9.0 [3.0; 20.0]	10 [5.7; 20.0]	− 2.5 (− 8.0; 3.0)	0.38
Ventilation duration to D28 for recipients of 3 UC-MSC doses	11.0 [6.0; 24.0]	13.0 [7.0; 22.0]	− 0.5 (− 8; 6)	0.79
SOFA-score change D0–D7	− 1.5 [− 2; 0.75]	− 2 [− 3.2; 0.2]	0.5 (− 2.0; 3.0)	0.60
SOFA-score change D0–D14	− 0.5 [− 1.2; 1.0]	− 3.0 [− 3; − 1.0]	1.5 (− 1.0; 5.0)	0.12
Organ-failure–free days to D14, *n*	3.0 [0; 6.0]	2.0 [0; 9.0]	− 0.5 (− 4.0; 3.0)	0.96
Organ-failure–free days to D28, *n*	16.0 [2.0; 20.0]	15.0 [0.75; 23]	− 0.5 (− 7.0; 4.0)	0.68
Days to reach PaO_2_/FiO_2_ > 200	5.0 [0; 16.0]	2.5 [0; 6.5]	0.74 [0.3; 1.6]	0.44
Days to reach PaO_2_/FiO_2_ > 300	12.0 [7.0; 23.0]	15.0 [5.0; 27.0]	1.1 [0.5; 2.3]	0.87
Days to ICU discharge^a^	15.0 [8.0; NA]	13.0 [5.5; 27]	0.8 [0.4; 1.7]	0.59
Days to weaning^b^	13.0 [9.0; NA]	17.0 [8.0; NA]	1.3 [0.5; 3.3]	0.55
Compliance change D0–D7	− 3.6 [− 11.8; 4.7]	− 0.1 [− 4.5; 2.4]	0.4 (− 24.7; 25.5)	1
Compliance change D0–D14	− 3.0 [− 3.0; − 3.0]	2.5 [− 0.9; 11.7]	− 5.52 (− 35.4; 1.1)	0.8
Driving pressure change D0–D7	0.5 [− 3.2; 4.2]	0.5 [− 1.2; 2.2]	0 (− 13.0; 13.0)	1
Driving pressure change D0–D14	1.0 [1.0; 1.0]	− 1.5 [− 2.0; 0.2]	2.5 (− 3.0; 3.0)	0.8
D28 mortality	5.0 (26.3%)	4.0 (18.2%)	2.0 (0.5; 8.5)	0.36

Values are expressed as median [interquartile range], or number (%), unless stated otherwise

*CI* confidence interval, *D* day, *HR* hazard ratio, *ICU* intensive care unit, *NA* not applicable, *PaO*_*2*_*/FiO*_*2*_ ratio of partial pressure of oxygen to fractional inspired oxygen, *PEEP* positive end-expiratory pressure, *SOFA* Sepsis-related Organ-Failure Assessment, *UC-MSCs* umbilical cord-derived mesenchymal stromal cells

^a^Censored at day of death for patients having died before D28 and censored at D28 for those patients still in the ICU at D28

^b^Estimated for the subgroup of patients ventilated at randomization, i.e., 31 patients (11 in CSM-CO group and 20 in placebo group). At Day 28, 6 had died (and censored at time of death), 17 were weaned, and 8 were alive and not weaned yet (censored at D28)

Sensitivity, per-protocol, and subgroup analyses (Additional file 1: Table S6) showed similar results.

**Fig. 2:**
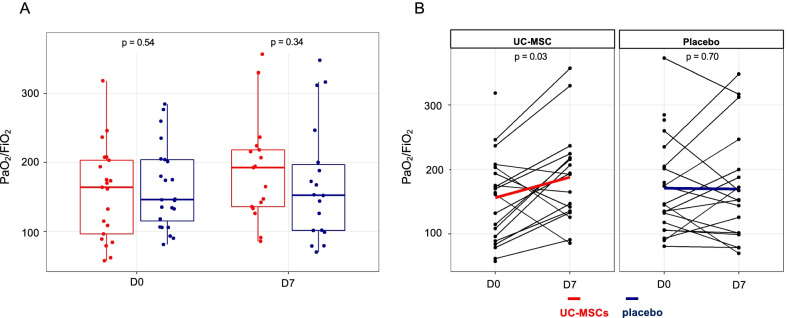
Primary endpoint: PaO_2_/FiO_2_ values and their changes between days 0 and 7. **A** Baseline (D0) and D7 PaO_2_/FiO_2_ ratios were similar for the two groups. Box plots of PaO_2_/FiO_2_ ratios: internal *horizontal lines* are the medians; *lower* and *upper box limits* are the 25th–75th interquartile range, respectively; and vertical bars represent the 10th and 90th percentiles. **B** PaO_2_/FiO_2_ ratios increased significantly from D0 to D7 in the UC-MSC group (respectively, 156.2 ± 68.2 vs 188.3 ± 74.2; Wilcoxon signed-rank exact test). The placebo group’s PaO_2_/FiO_2_ ratios on D0 and D7 were comparable (respectively, 171.2 ± 72.9 vs 169.8 ± 85.6, Wilcoxon signed-rank exact test). UC-MSCs group in *red*; placebo group in *blue*. *D* day. *PaO*_*2*_*/FiO*_*2*_ ratio of partial pressure of oxygen to fractional inspired oxygen. *UC-MSCs* umbilical cord-derived mesenchymal stromal cells

Although the D0 PaO_2_/FiO_2_ ratios for the two groups were similar (Fig. 2A), the UC-MSC group’s ratio increased significantly from D0 to D7 (156.2 ± 68.2 vs 188.3 ± 74.2, respectively; *P* = 0.03) for the subgroup of patients remaining alive in the ICU over the first 7 days (Fig. 2B), but the numerical difference was no longer significant according to the post-imputation analysis (156.2 ± 68.2 vs 194.7 ± 95.3, respectively; *P* = 0.08). In contrast, the placebo group’s PaO_2_/FiO_2_ ratio remained unchanged from D0 to D7.

### Secondary endpoints

No significant between-group secondary-endpoint differences were observed for SOFA scores, PaO_2_/FiO_2_ ratios, compliance, driving pressure change between D0 and D7 or D14, organ-failure–free days, ventilation-free days, duration of ventilation, time to weaning, time to ICU discharge, time to reach PaO_2_/FiO_2_ > 200 or > 300, and mortality to D28 (Table 4 and Additional file 2: Figure S1).

Before and after D14, the numbers of adverse events, serious adverse events, and patients with any adverse and/or any serious adverse events were similar for the two groups (Table 5, Additional file 1: Table S7). In total, 36 (80%) patients experienced adverse events prior to D14, and 18 (40%) thereafter. The vast majority of adverse events were considered to be related to COVID-19 progression. Only one UC-MSC-group patient experienced an adverse event (diarrhoea) deemed possibly related to the study treatment. Only one placebo patient suffered a pre-specified adverse hemodynamic event within 6 h of infusion onset (Additional file 1: Table S8). Six (28.6%) UC-MSC recipients had pre-formed DSA, i.e., present before treatment and directed against the HLA of the infused UC-MSCs received. On D14, three (14.3%) patients had synthesized de novo low-level DSA. Eleven (45.8%) placebo recipients had performed anti-HLA, and no patient developed de novo significant anti-HLA immunization between D0 and D14.

**Table 5:** Summary of the 139 reported adverse events occurring in the 24 UC-MSC- and 21 placebo-treated patients

	UC-MSC	Placebo	Total	*p* value
Adverse events D0–D14				
Subjects with AEs	18/21 (85.7%)	18/24 (75%)	36/45 (80%)	0.47
AEs reported	49/97 (50.5%)	48/97 (49.5%)	97 (100%)	0.29
Subjects with SAEs	6/21 (28.6%)	6/24 (25%)	12/45 (26.7%)	0.79
SAEs reported	10/49 (20.4%)	6/48 (12.5%)	16/97 (16.5%)	0.29
AEs by severity				0.99
Mild	16/49 (32.7%)	15/48 (31.3%)	31/97 (32%)	
Moderate	24/49 (49%)	24/48 (50%)	48/97 (49.5)	
Severe	9/49 (18.4%)	9/48 (18.7%)	18/97 (18.6)	
AE grade^a^				0.14
Grade 1	19/47(40.4%)	10/48 (20.8%)	29/95 (30.5%)	
Grade 2	15/47 (31.9%)	19/48 (39.6%)	34/95 (35.8%)	
Grade 3	9/47 (19.1%)	16/48 (33.3%)	25/95 (26.3%)	
Grade 4	4/47 (12.5%)	3/48 (6.3%)	7/95 (7.4%)	
AEs by treatment relatedness				0.41
Possible	1/48 (2.1%)^b^	0 (0%)	1/95 (1.1%)	
Other treatment	4/48 (8.3%)	2/47 (4.3%)	6/95 (6.3%)	
Other disease	1/48 (2.1%)	1/47 (2.1%)	2/95 (2.1%)	
COVID-19 progression	28/48 (58.3%)	37/47 (78.7%)	65/95 (68.4%)	
Other causes	3/48 (6.3%)	2/47 (4.3%)	5/95 (5.3%)	
Undetermined	11/48 (22.9%)	5/47 (10.6%)	16/95 (16.8%)	
Adverse events after D14				
Subjects with AEs	9/21 (38.1%)	9/24 (37.5%)	18/45 (40%)	0.71
AEs reported	19/42 (45.2%)	23/42 (54.8%)	42 (100%)	1.00
Subjects with SAEs	4/21 (19%)	4/24 (16.7%)	8/45 (17.8%)	1.00
SAEs reported	4/19 (21.1%)	4/23 (17.4%)	8/42 (19%)	1.00
AEs by severity				0.19
Mild	7/19 (36.8%)	3/23 (13%)	10/42 (23.8%)	
Moderate	7/19 (36.8%)	12/23 (52.2%)	19/42 (45.2%)	
Severe	5/19 (26.3%)	8/23 (34.8%)	13/42 (31%)	
AE grade^a^				0.18
Grade 1	7/18 (38.9%)	3/23 (13%)	10/41 (24.4%)	
Grade 2	7/18 (38.9%)	15/23 (65.2%)	22/41 (53.7%)	
Grade 3	1/18 (5.6%)	3/23 (1%)	4/41 (9.8%)	
Grade 4	3/18 (16.7%)	2/23 (8.7%)	5/41 (12.2%)	
AEs by treatment relatedness				0.54
Possible	0 (0%)	0 (0%)	0 (0.0%)	
Other treatment	3/14 (21.4%)	4/20 (20%)	7/34 (20.6%)	
Other disease	1/14 (7.1%)	0 (0%)	1/34 (2.9%)	
COVID-19 progression	8/14 (57.1%)	11/20 (55%)	19/34 (55.9%)	
Other causes	2/14 (14.3%)	2/20 (10%)	4/34 (11.8%)	
Undetermined	0 (0%)	3/20 (15%)	3/34 (8.8%)	

Values are expressed as number (%). *AEs* adverse events. *SAEs* severe adverse events. *UC-MSC* umbilical cord-derived mesenchymal stromal cells

^a^Grade from the Common Terminology Criteria for Adverse Events classification

^b^Possible non-serious treatment-related AE: diarrhoea

Inflammatory biomarker-analysis (Additional file 3: Figures S2 and Additional file 4: Figure S3) and plasma virus-load results (Additional file 5: Figure S4) are reported in Additional file 1. Later decreases of inflammatory markers in the UC-MSC-treated group were the only significant difference found (Additional file 3: Figure S2).

## Discussion

The main finding of this study is that repeated intravenous infusions of UC-MSCs into patients with SARS–CoV-2-induced early ARDS were safe, but did not improve oxygenation, as reflected by the PaO_2_/FiO_2_-ratio change between D0 and D7, compared to the placebo group.

Two randomized trials have previously tested UC-MSCs in COVID-19 patients [30]. Whereas the study by Lanzoni et al. [30] included patients with ARDS and/or hypoxemia with SpO2 < 94%, the patients enrolled by Dilogo et al. [31] were those with leukopenia and severe COVID-19 pneumonia, without further specification. Although the percentage of patients on invasive mechanical ventilation at inclusion was given in one study [30], neither of the two studies reported the distribution of invasively ventilated patients and their differences in UC-MSC and placebo groups, either at baseline or during follow-up. While the majority of patients described by Lanzoni et al. were also receiving adjuvant treatments administered at inclusion (i.e., remdesivir, convalescent plasma, tocilizumab, corticosteroids or hydroxychloroquine), this information was not given in the second study [31]. Finally, while the MSC-administration scheme consisted of two infusions of 100 million cells at day D0 and D3 for the former study, the latter administered one million/kg in a single infusion given between D2 and D30. The number of cell passages before use was not indicated in either study.

Our double-blind, randomized, placebo-controlled trial has several other key strengths, including serial determinations of a wide array of inflammation and immunity-related biomarkers at several times until D14, monitoring of allo-immunization, and thorough characterization of the final UC-MSC-based product. Instead of assessing the three-lineage differentiation of our cells, we rather used a potency assay more specific for their intended immunomodulatory effect and to this end performed mixed lymphocyte reactions which confirmed their inhibitory effect on allogeneic T lymphocytes [34]. A final key strength is the multicenter design. However, the small number of patients included in ten centers over 6 months has several origins: (1) the pandemic dynamics were in successive waves, with periods of acceleration and deceleration of patient flows; (2) many patients admitted to the ICU were already included in phase 2 or 3 therapeutic trials, precluding inclusion in our trial; (3) some patients could not be included because of the unavailability of the manufacturing cell therapy unit. For the future, scaling up MSC production made possible by current technologies should allow to cover the needs of large numbers of patients.

A second important finding is that repeated UC-MSC infusions were not associated with any serious adverse events during treatment or thereafter (until D28). More specifically, data collected from continuous hemodynamic and respiratory monitoring during intravenous infusion of UC-MSC suggested good clinical tolerance during UC-MSC administration, with no transfusion incompatibility or infusion-related events. MSCs have already been used to treat a wide variety of diseases without safety issues, but those results might not be directly applicable to COVID-19, because one of the disease’s hallmark is the potential for pulmonary thrombotic microangiopathy, which could have been worsened by UC-MSCs obstructing the pulmonary capillary bed, thereby causing right heart failure. No such event was documented in our trial. At least three factors might have contributed to the good tolerance of UC-MSC infusions: 1) when MSCs are cultured in 5% human-platelet lysate and for no longer than 3–4 passages, as in our trial, the reported cell size (in suspension) had been ~ 17 μm, which should keep cells within the safety range in terms of risk of vascular obstruction [35]; (2) the intrinsic prothrombotic activity of UC-MSCs is mitigated by intermediate-level anticoagulation administration, now recommended and widely given to hospitalized COVID-19 patients [2], and it is reassuring that sCD40L levels, known to increase coagulation, were not elevated in UC-MSC-treated patients compared with controls (data not shown); and (3) the rapid apoptotic fragmentation of intravenously delivered UC-MSCs that became trapped in lung tissue and underwent progressive size diminution [36], and subsequent phagocytosis of apoptotic fragments by monocytes and neutrophils [37] could have contributed further to cell clearance from the vascular bed. Finally, it is noteworthy that only three of the UC-MSC-treated patients developed allo-immunization to the infused cells and their mean fluorescence intensity (MFI) values were in the low range, unlikely to be cell-damaging [38]. That finding is consistent with previously reported clinical study results documenting the low immunogenicity of UC-MSCs [39].

Concerning efficacy, the trial did not meet its primary endpoint, since PaO_2_/FiO_2_-ratio changes between D0 and D7 did not differ significantly between UC-MSC– and placebo-infused patients. First, this lack of difference might suggest that, when COVID-19-associated respiratory failure is severe enough to warrant invasive mechanical ventilation or ventilatory support, the extent of lung damage outweighs the effects of UC-MSCs, at least when delivered according to the dosing schedule used herein, and thus their capacity to promote lung-tissue repair translating into improved oxygenation patterns. That lack of efficacy contrasts with the spectacularly improved survival obtained in two previous trials [30, 31] that applied a similar double-blind, randomized design. However, in those studies, control-group mortality was unusually high (50% [31] to 58% [30] vs 21.9% herein), which allowed room for clearer demonstration of treatment effects. Furthermore, in both studies, although invasively ventilated patients were included, their distributions in each group were not specified; severity scores were missing and the timing of UC-MSC administration in relation to the ARDS stage (early or late) was either focused on the early [30] or the resolution phase (i.e., > 7 days) of ARDS [31], thereby making comparisons between the studies difficult. Second, the presence of pre-formed and de novo DSA in six (28.6%) and three (14.3%) UC-MSC-treated patients, respectively, might have altered therapeutic efficacy by accelerating MSC clearance, but this is unlikely because these patients’ MFI levels were low-to-moderate and, except for one, remained below the 5,000 threshold level beyond which DSA-HLA-1 MFIs correlate with complement-dependent and antibody-dependent cell-mediated cytotoxicities [40]. The choice of the primary endpoint is a third possible explanation. The absence of FiO_2_ or positioning (supine or prone) standardization for measuring PaO_2_/FiO_2_ might have increased the variability of its values from D0 to D7 [41], thereby hindering a beneficial or deleterious effect evaluated in the study. Moreover, some authors have questioned the prognostic value of PaO_2_/FiO_2_ in ARDS patients [42]. The PaO_2_/FiO_2_ ratio has several limitations [43]: (1) it does not reflect the ventilation applied and does not account for mean airway pressure or positive end-expiratory pressure; (2) it is dependent on barometric pressure; (3) it cannot distinguish hypoxemia due to alveolar hypoventilation from other causes, such as ventilation-perfusion mismatch and shunt; (4) it is markedly dependent on FiO_2_; (5) it is highly dependent on oxygen extraction capacity; (6) it does not indicate oxygen content of the blood, or oxygen delivery to tissues. On the other hand, the study was designed at the very beginning of the first French pandemic wave, in February 2020, and, at that time, locally available data (from several ICUs in Paris) suggested a potentially notable improvement of survivors’ gas exchange on D7 (no published or quantified data available at that time). Therefore, we decided to use the day-7 PaO_2_/FiO_2_ ratio as the primary endpoint. Another hypothesis explaining the absence of therapeutic effects is a potential lack of power of the study. Indeed, most phase 2 studies on ARDS had wide confidence intervals that, most often, limited generalizability [44]. Thus, our finding that the UC-MSC-group’s—but not the placebo group’s—PaO_2_/FiO_2_ ratio rose significantly from D0 to D7 might have reflected some treatment efficacy that remained too modest to emerge with our sample size. However, this observation must be balanced against the fact that: (1) the numerical increase of the UC-MSC group’s-PaO_2_/FiO_2_ ratio from D0 to D7 was no longer significant following the post-imputation analysis, and (2) more placebo-group patients were invasively ventilated, under vasopressors and neuromuscular blocking agents, thereby indicating greater disease severity and potentially favoring the treated group.

Concerning circulating biomarker-level changes, it remains unclear whether the UC-MSC group’s later appearance of the proinflammatory cytokine- and chemokine-level declines is a signature of the treatment, potentially leading to any benefit in terms of inflammation resolution. Results of previous randomized-controlled studies showed either a trend [31] or a significant immunoregulatory effect [30], with UC-MSC-treated patients having marked reductions of several circulating inflammatory biomarkers from D0 to D6–D7. Those heterogeneous findings can probably be explained by the differences in therapeutic doses, timing, choices of biomarkers to be measured and the small numbers of patients included, but also by the fact that those marker levels were determined in plasma and not the alveolar compartment. Indeed, Wick et al. recently found the MSC immunomodulatory effect in non-COVID-19 patients with ARDS to be more detectable in bronchoalveolar lavage at 48 h post-MSC administration than in blood [45]. In future studies exploring the immunomodulatory impact of MSCs in patients with COVID-19-associated ARDS, it will probably also be important to measure these markers at early time points post-infusion and in bronchoalveolar lavage, if feasible.

We acknowledge that the STROMA–CoV-2 trial has limitations. They include the small sample size and the lack of a robust sample-size calculation explained by the paucity of published data at that time on the distribution and kinetics of PaO_2_/FiO_2_ ratio in this patient population; the likely patient-management modifications clearly illustrated by an almost systematic use of corticosteroids during the second wave of the pandemic, with the caveat that, although in vitro study results indicated a generic cytotoxic effect of these drugs on MSCs, dexamethasone, which has been the gold standard during the pandemic (and was used in our trial), is the one with the least harmful effects on cell viability [46]; the higher percentage of invasively ventilated placebo-group patients on neuromuscular blocking agents and vasopressors, probably explained in part by the randomization-ratio change introduced between the 1st and 2nd pandemic waves, may have favored the treated group. However, the inclusion SOFA score, lung injury score, and PaO_2_/FiO_2_ ratio were comparable for the two groups; potential differences might exist in the bioactivities of the infused cells, despite quality-control consistency between the first and second batches, derived from 2 distinct donors; and the dosing and timing of delivery schedule are still debated. Indeed, the UC-MSC doses used have varied across studies, with a median dose of 100 million for intravenous delivery and a minimal effective dose ranging from 70 to 190 million/patient/dose [47]. Furthermore, although we are not aware of any comparison of a total aggregate dose given as a single bolus versus its fractionation over time, the rapid UC-MSC clearance led us to adopt repeated dosing, in hopes of inducing a longer-term effect. Those considerations rationalized our choice of three 1 × 10^6^ UC-MSCs/kg/dose repeated every other day, a dosing strategy consistent with several of the registered cell-therapy trials on COVID-19 patients [48]. However, it remains to be assessed whether outcomes can be improved by higher doses and/or longer time intervals between cell deliveries.

## Conclusions

The results of this phase 2b, multicentre, double-blind, randomized, placebo-controlled trial showed no efficacy of human UC-MSCs on the PaO_2_/FiO_2_-ratio change between D0 and D7 in patients with SARS–CoV-2-induced ARDS compared to placebo. D28 mortality also did not differ. Despite the lack of statistically significant differences, UC-MSC-treated patients’ greater PaO_2_/FiO_2_-ratio increase between D0 and D7, compared to placebo-infused controls, might represent a signal warranting further investigation on a larger patient population. Repeated UC-MSC infusions were not associated with any serious adverse events during treatment or thereafter (until D28). Consequently, to better assess in which direction this treatment strategy shifts the risk–benefit and cost-effectiveness balances, pursuit of this avenue of research on COVID-19-associated pneumonia would be notably enhanced by greater homogeneity of patient demographics and standardized therapeutic protocols, reappraisal of the most clinically relevant endpoints and larger sample sizes.

## Supplementary Information

**Additional file 1:** A detailed description of the methods and the full clinical trial protocol. **Table S1.** Quality-control characteristics of UC-MSCs used as treatment: identity and safety. **Table S2.** Quality-control characteristics of batch-1 UC-MSCs used as treatment: identity and safety. **Table S3.** Quality-control characteristics of batch-2 UC-MSCs used as treatment: identity and safety. **Table S4.** Evolution of respiratory characteristics from baseline (D0) to D28 **Table S5.** UC-MSCs posology per patient. **Table S6.** Subgroup analyses of the primary outcome. **Table S7.** Complete list of adverse events. **Table S8.** Summary of pre-specified infusion-associated adverse events for randomized subjects.

**Additional file 2: Figure S1.** Survival probabilities and SOFA scores. (A) Survival rates from D0 to D28 were comparable for the two groups (P = 0.63, log-rank test). (B) SOFA-score evolutions from D0 to D28 did not differ (P = 0.79, Wilcoxon test). Data are expressed as mean per day ± standard deviation. *D* day. *SOFA* Sequential Organ-Failure Assessment score. *UC-MSCs* umbilical cord-derived mesenchymal stromal cells.

**Additional file 3: Figure S2.** Analysis of plasma inflammatory cytokines, chemokines, growth factors, and biomarkers at baseline (D0) and D2, D4, D7, and D14 after starting infusions. Concentrations of 48 cytokines were quantified in plasma from UC-MSC– (*n* = 20) or placebo- treated (*n* = 21) patients. Statistical analyses with Wilcoxon rank-sum tests compared values between groups (A) and within each group (B and C) at each day indicated. Volcano plots were generated for each comparison to show the log_2_ fold-changes relative to placebo or D0, with statistical significance reported as −log_10_ *P*-values. Significance, defined as *P* < 0.05, is in *blue,* with those remaining statistically significant after multiple corrections (Benjamini–Hochberg correction) in *red D* day, *D0* baseline, *UC-MSC* umbilical cord-derived mesenchymal stromal cell, *ANGP-1* angiopoietin-1, *BCA-1* B-cell-attracting chemokine-1, *CTACK* cutaneous T-cell-attracting chemokine, *CXCL-9* chemokine (C-X-C motif) ligand 9, *EGF* epidermal growth factor, *FLT-3L* Fms-related tyrosine kinase-3 ligand, *G-CSF* granulocyte-colony-stimulating factor, *IFN-γ* interferon-γ, *IL* interleukin, *IP-10* interferon gamma-induced protein-10, *MCP* monocyte chemoattractant protein, *M-CSF* macrophage-colony-stimulating factor, *MDC* macrophage-derived chemokine, *PDGF-AA* platelet-derived growth factor-AA, *RAGE* receptor for advanced glycation end products, *sCD40L* soluble cluster of differentiation-40 ligand, *SDF* stromal cell-derived factor, *SPD* surfactant protein B, *TGF-α* transforming growth factor-α, *TPO* thrombopoietin, *VEGF-A* vascular endothelial growth factor-A.

**Additional file 4: Figure S3.** Analysis of plasma inflammatory cytokine, chemokine, growth factor, and biomarker concentrations on D0 (baseline), D2, D4, D7, and D14 after starting infusions. The figure reports the quantification results for 10 cytokines selected among the 48 sought in plasma samples from patients treated with UC-MSCs (*n* = 20) or placebo (*n* = 21). Data are log_2_ transformed. Box plots of PaO_2_/FiO_2_ ratios: internal *horizontal lines* are the medians, *lower* and *upper box limits* are the 25th and 75th interquartile range, respectively, vertical bars are drawn down to the 10th percentile and up to the 90th percentile. *D* day, *IL* interleukin. *IP-10* interferon-gamma-induced protein-10, *MCP* monocyte chemoattractant protein, *RAGE* receptor for advanced glycation end products, *SDF-1* stromal cell-derived factor-α, *UC-MSCs* umbilical cord-derived mesenchymal stromal cells.

**Additional file 5: Figure S4.** Analysis of plasma SARS–CoV-2 RNA and N-antigenemia levels at baseline (D0) and D2, D4, D7, and D14 after starting infusions. Plasma SARS–CoV-2 RNA (by RT-PCR) and N-antigenemia in UC-MSC– (*n* = 21, red) or placebo-treated (*n* = 24, blue) patients were quantified. Based on viral RNA levels (A), five (23.8%) UC-MSC– and eight (33.3%) placebo-treated patients had detectable viremia on D0, while (B) N-antigenemia was positive for 20 (95.2%) and 22 (91.7%) patients, respectively. (C) Plasma SARS–CoV-2 NAg-level change from D0 to D14. Data are expressed as mean ± standard deviation. The percentage of viremic patients and N-antigenemia levels decreased sharply until D4 (A–C). No between-group difference was observed in terms of percentage of viremic patients and/or decline from D0 to D14. Red = UC-MSC group; blue = placebo group. *D* day, *PaO*_*2*_*/FiO*_*2*_ ratio of partial pressure of oxygen to fractional inspired oxygen, *RT-PCR* reverse transcription-polymerase chain reaction, *SARS–CoV-2* severe acute respiratory syndrome coronavirus-2, *UC-MSCs* umbilical cord-derived mesenchymal stromal cells.

## Data Availability

Qualified clinical researchers can request access to de-identified participant dataset, informed consent forms and related documents, including the study protocol that underlie this article through submission of a proposal with a valuable research question to the corresponding author, subject to agreement of a contract.

## Abbreviations

ANCOVA: Analysis of covariance
ARDS: Acute respiratory distress syndrome
CFU: Colony-forming unit
COVID-19: Coronavirus disease-2019
D: Day
DSAs: Donor-specific anti-human leukocyte antigen antibodies
PaO_2_/FiO_2_: Partial pressure of oxygen to fractional inspired oxygen
HLA: Human leukocytes antigen
ICU: Intensive care unit
IL: Interleukin
IQR: Interquartile range
LOCF: Last Observation Carried Forward
MFI: Mean fluorescence intensity
MSCs: Mesenchymal stromal cells
RT-PCR: Reverse transcriptase-polymerase chain reaction
SARS-COV-2: Severe acute respiratory syndrome coronavirus-2
SOFA: Sequential Organ-Failure Assessment
UC: Umbilical cord
