# Supplementary material for: Treatment of COVID-19-associated ARDS with mesenchymal stromal cells: a multicenter randomized double-blind trial
Source: Crit Care. 2022 Feb 21;26:48. doi: 10.1186/s13054-022-03930-4 (PMC8860258; doi:10.1186/s13054-022-03930-4)
Supplement: Supplementary file 1 — Additional file 1: A detailed description of the methods and the full clinical trial protocol. Table S1. Quality-control characteristics of UC-MSCs used as treatment: identity and safety. Table S2. Quality-control characteristics of batch-1 UC-MSCs used as treatment: identity and safety. Table S3. Quality-control characteristics of batch-2 UC-MSCs used as treatment: identity and safety. Table S4. Evolution of respiratory characteristics from baseline (D0) to D28 Table S5. UC-MSCs posology per patient. Table S6. Subgroup analyses of the primary outcome. Table S7. Complete list of adverse events. Table S8. Summary of pre-specified infusion-associated adverse events for randomized subjects. [file 13054_2022_3930_MOESM1_ESM.docx]

**Treatment of COVID-19-associated ARDS with mesenchymal stromal cells: a multicenter randomized double-blind trial.**

Antoine Monsel

Caroline Hauw-Berlemont

Miryam Mebarki

Nicholas Heming

Julien Mayaux

Otriv Nguekap Tchoumba

Jean-Luc Dieh

Alexandre Demoule

Djillali Annane

Clémence Marois

Sophie Demeret

Emmanuel Weiss

Guillaume Voiriot

Muriel Fartoukh

Jean-Michel Constantin

Bruno Mégarbane

Gaëtan Plantefève

Stéphanie Mallard-Castagnet

Sonia Burrel

Michelle Rosenzwajg

Nicolas Tchitchek

Hélène Boucher-Pillet

Guillaume Churlaud

Audrey Cras

Camille Maheux

Chloé Pezzana

Mamadou Hassimiou Diallo

Jacques Ropers

Philippe Menasché

Jérôme Larghero

on behalf of the APHP STROMA–CoV-2 Collaborative Research Group

**Additional File 1**

**METHODS**

**Patients and Exclusion Criteria**

Eligible patients had Berlin criteria-defined ARDS for <96 hours, reverse transcriptase–polymerase chain reaction (RT-PCR)-confirmed SARS–CoV-2 infection, and were receiving respiratory support (invasive or non-invasive mechanical ventilation, and/or high-flow nasal oxygenation), with positive end-expiratory pressure (PEEP) equivalent ≥5 cm H2O. The need for high-flow nasal oxygen therapy was sufficient to define "ventilatory support" status, regardless of the gas flow and FiO_2_ parameters. Exclusion criteria included age <18 years, acute respiratory distress syndrome (ARDS) present for >96 hours, pulmonary fibrosis, pulmonary hypertension (WHO classification class III or IV), pulmonary embolism within the previous 3 months, extracorporeal membrane oxygenation or life support, immunocompromised status **(**i.e., constitutional like human immunodeficiency virus, or drug-induced with chronic intake of immunosuppressant(s) or corticosteroids at an immunosuppressive dose for >1 month (≥10 mg/day of prednisolone equivalent)), pregnancy or breastfeeding, treatment for cancer in the past 2 years, an underlying medical condition with life expectancy <6 months, moderate-to-severe liver disease (Child–Pugh score >12), severe chronic lung disease with the use of home oxygen, or partial arterial pressure of carbon dioxide >50 mm Hg. Patients not committed to full support (ie, had do not resuscitate or limit life support orders) were also excluded. Written informed consent was obtained from patients or a legally designated representative. When the latter was absent, the investigator decided to include the patient because of the therapeutic emergency. In that case, the patient’s or representative’s consent for continuation of the protocol was to be obtained as soon as possible.

**Randomization and Blinding**

Randomization and data collection were centralised using a web-based electronic case-report form (Cleanweb Telemedicine Technologies™) on a sponsor-operated secure server.

To maintain blinding of the investigators and clinicians, conditioning, labelling and distributions of UC-MSCs and placebo pouches were rigorously identical, with both being pre-conditioned in opaque bags. The intravenous infusion tubing set was not blinded *per se*. Only the bag was covered. However, it has been considered that, given the dilution of the cell suspension, and, thus, the transparent appearance of the fluid, it was not possible for the treating staff to ascertain whether or not the patient received the treatment or the placebo only by looking at the infusion set. Investigators remained unaware of treatment allocations until the database was fully cleansed and exported for statistical analysis.

**Production and Characterisation of UC-MSC–based Advanced Therapy Medicinal Product (ATMP)**

The experimental treatment is a suspension of allogenic mesenchymal stromal cells isolated from human umbilical cord Wharton’s jelly (UC-MSCs). Two UCs were collected from two healthy donors, according to the European Directive 2004/23/EC, after written informed consents had been obtained and serological testing done according to national regulatory agency requirements. At the end of the production process, the two cell populations were used separately., *i.e*., each patient received UC-MSC doses from a single umbilical cord. Full-term delivery cords were collected and processed within 24 hours. The active substance was produced at the Saint-Louis Hospital Cell Therapy Unit of Paris and the ATMP was produced at the MEARY Cell and Gene Therapy Center, according to good manufacturing practices (GMPs). Briefly, MSCs were isolated from UCs by enzymatic digestion or the explant method, then expanded in complete culture medium composed of Nutristem® MSC XF Basal Medium (Biological Industries, Ref 05-200-1A) + Nutristem® MSC XF Supplement Mix (Biological Industries, Ref 05-200-1U) + 5% irradiated platelet lysate MultiPL’100i (Macopharma, Ref BC0190032) + sodium heparin 2 IU/mL (Panpharma, Ref 5520508). Cells were maintained at 37°C in a humidified atmosphere with 5% CO_2_ and the culture medium was changed twice a week. UC-MSCs were expanded until passage 4 maximum, harvested using a Recombinant Trypsin-EDTA Solution (Biological Industries, Ref 03-079-1B), and cryopreserved in a 10% dimethyl sulfoxide solution at a mean (±SD) dose of 100±10×10^6^ UC-MSCs per bag.

Quality controls of the experimental ATMP included cell concentration, identity, and purity (immunophenotype), functionality (viability, clonogenicity, and immunosuppressive effects assessed by mixed lymphocyte reaction [MLR]), and safety (microbiological sterility, endotoxins, and mycoplasma assays with a posteriori results; karyotype). Cell number was determined using the Nucleocounter NC 200® (ChemoMetec, Denmark). The UC-MSC phenotype was determined by flow cytometry (MacsQuant10, Miltenyi), using the surface antigens CD105, CD73, CD90, CD45, CD34, CD11b, CD19, and HLA-DR from the BD stemflow hMSC Analysis Kit (BD Biosciences, ref 562245). Cell viability was evaluated by using the eBioscience Fixable Viability Dye eFluor 780 assay (Invitrogen, ref 65-0865). Cell clonogenicity was assessed using the colony-forming–unit-fibroblast (CFU-F) assay.

To assess UC-MSC immunomodulatory properties, ie, evaluate their inhibitory effect on T-cell proliferation in vitro, MLRs were run, as described by Nicotra et al (1). Our results showed that UC-MSCs significantly inhibited T-cell proliferation by 86±5% for a UC-MSC/PBMC ratio of 1:1. Pertinently, that inhibitory effect was dose-dependent, decreasing proportionally to the UC-MSC/PBMC ratio: (78±10% at ratio 1:3; 77±10% at ratio 1:10, 70±3% at ratio 1:30, 44±20% at ratio 1:100 and 18±15% at ratio 1:300). Microbiological testing used aerobic and anaerobic BacT/ALERT® (BioMerieux, ref 259789/259790) in BacTALERT® VIRTUO® Microbial Detection System (BioMerieux). Endotoxins were quantified on Multiskan Sky Spectrophotometer (ThermoFisher Scientific) using Chromo-LAL® reagents (Associates of Cape Cod, ref C0031) and mycoplasma on QuantStudio 5 Real-Time PCR System (ThermoFisher Scientific) using Venor® GeM qEP Kit (Minerva Biolabs, ref 11-9250). The karyotype was determined before MSC cryopreservation in a certified laboratory (Cerba, France) on a minimum of 20 metaphases.

The day of patient treatment, one bag was thawed. The collected cells were washed and underwent a final quality control before being suspended at the dose of 10^6^ cells/kg in a final volume of 150 mL of 0∙9% NaCl/0∙5% albumin. The maximum dose was set at 80×10^6^ cells per infusion for the following reasons: 1) fitting a reasonable theoretical average weight; 2) controlling the risk of pulmonary capillary trapping; 3) making the protocol compatible with the capacity to produce UC-MSCs. Moreover, this dose was repeated 3 times over 5 days in our protocol, making the maximum total dose was 240 million cells per patient, which remains well above the median therapeutic dose of 100 million cells per patient described in the literature. The final product was released according to the following criteria and specifications: 10^6^ UC-MSCs/kg dose, viability ≥70%, identity markers matching a typical MSC phenotype. Finally, the pouch was transferred to the ICU at 18–24°C, within 4 hours after thawing. The placebo was a solution of 150 mL of 0∙9% NaCl. A single manufacturing center (production and quality control) provided cells for all investigating centers. All the thawing, washing and dose adjustment steps were done in a dedicated clean room at MEARY Center (SLS – Saint Louis Hospital), with a Quality-Control Laboratory located on the same floor of the building. Product-expiration time was determined from the final bag-sealing step (the 4-hour period did not include the thawing/washing steps).

The experimental ATMP was not stored before administration. After release, the product was immediately shipped to the pharmacist of the participating centers who brought it immediately to the ICU for administration. The bag was transported to centers at +18 to +24 ° C in a sealed container by an approved carrier in an average time of 59 min (TNN: 55 min; BJN: 63 min; PSL: 39 min; HEGP: 48 min; RPC: 74 min, ARG: 92 min; LRB: 42 min). The infusion could start within 4 hours following ATMP-manufacturing completion (weighing and final sealing of the bag), i.e., the infusion duration period was not included in this 4-hour product expiration time.

**Circuit of the UC-MSC-based ATMP**

After its release, the experimental ATMP or the placebo was transferred to the pharmacy of each recruiting center, with instructions for use and traceability documents. After receipt and control, the pharmacy distributed the treatment pouches to the ICU. The experimental ATMP/placebo was administered to the patient by slow intravenous perfusion, within 4 hours after thawing. UC-MSCs or placebo treatments were infused intravenously by drip over 60 min through a standard blood-filter tubing set (pore size 170 μm).

**Clinical and Biological Outcomes**

Secondary endpoints were all-cause mortality at D28, number of ventilator-free days to D28, duration of ventilation in patients alive on D28, number of intensive care-free days to D28, number of organ-failure–free days to D28, SOFA score, lung injury score, driving pressure, and respiratory lung compliance. Patients were considered weaned-off mechanical ventilation on the first day invasive mechanical ventilation was stopped, taken as the weaning date. The “days-to-weaning” duration corresponds to the time between the start of invasive mechanical ventilation and the first day without invasive mechanical ventilation. Safety endpoints were the number of adverse events attributable to UC-MSC administration, the number of pre-specified infusion-related adverse events (listed below) during the infusion of study treatments (ATMP or placebo), and the 6 hours following infusion onset, during the 24 hours following each administration on D1, D3 (±1 day) and D5 (±1 day), during the treatment period from D1 to D7, and any adverse events associated with UC-MSC infusion from D1 to D28. Pre-specified infusion-related adverse events from D0 to D7 could be assessed by continuous hemodynamic and respiratory monitoring (e.g., heart rate, blood pressure, oxygen saturation). The incidence and nature of all serious adverse events were reviewed periodically and independently assessed by the Data-Safety–Monitoring Board to determine whether they were related to UC-MSC administration, with special focus on events that would be unexpected in a critically-ill patient with ARDS.

**List of Prespecified Respiratory or Cardiovascular Infusion-Related Adverse Events**

During the infusion of study treatments (ATMP or placebo):

- A clinical picture compatible with a transfusion incompatibility or an infusion-linked event (urticaria, bronchospasm…)

During the 6 hours following infusion onset of study treatments:

- Onset of hemodynamic instability requiring administration of noradrenaline ≥2 mg/h; decreased blood pressure necessitating a higher dose of noradrenaline ≥2 mg/h compared to its initial value
- Ventricular tachycardia, ventricular fibrillation or cardiac arrest
- Development of severe hypoxia (partial pressure of oxygen to fractional inspired oxygen (PaO_2_/FiO_2_)) ≤60 mm Hg or ≥50% of its preinfusion value.

During the 24 hours following each infusion

- Any cardiac arrest.

During the treatment period from day (D) 1 to D7

- Massive pulmonary embolism with hemodynamic repercussions
- Deterioration of arterial oxygenation with severe hypoxia unexplained by the evolution of the SARS-CoV-2-induced ARDS and requiring implantation of venovenous-extracorporeal membrane oxygenation
- Any clinical evidence identified by the physician as unusual by its characteristics, frequency, or severity compared to the clinical evolution of ARDS.

**Analysis of Inflammatory Cytokines, Chemokines, Growth Factors and Biomarkers in Plasma at Baseline (D0) and D2, D4, D7, and D14 After Start of Infusions.**

The Luminex^®^-based multiplexed immunoassays with fluorescent microspheres (Milliplex® MAP assays-HCP2MAG-62K-PX23, TFGBMAG-64K-03, and HCYTA-60K-PX48) were used to quantify the plasma concentrations of 48 cytokines, chemokines, and growth factors. The epithelial and endothelial biomarkers keratinocyte growth factor (KGF), surfactant protein D (SPD), angiopoietin (ANGP-1 and -2), receptor for advanced glycation end products (RAGE) were measured using the following kits, respectively: Human KGF/FGF-7 Quantikine ELISA Kit#DKG00, Human SP-D Quantikine ELISA Kit#DSFPD0, Human Angiopoietin-1 Quantikine ELISA Kit#DANG10, Human Angiopoietin-2 Quantikine ELISA Kit#DANG20, and Human RAGE Quantikine ELISA Kit#DRG00 from R&D Systems®. All the analytes were quantified, according to the manufacturer’s protocol, with ELISA kits (Quantikine ELISA Kits, Bio-Techne SAS, Rennes, France) and Luminex assays (Milliplex, Millipore SAS, Sain-Quentin-en-Yvelines, France).

**SARS-CoV-2 Nucleocapsid-Antigenemia (N-Antigenemia) Assay**

Prior to analysis, plasma samples were stored at –80°C. N-Antigenemia levels were determined with the coronavirus disease (COVID)-Quantigene® CE-IVD ELISA microplate assay (AAZ, Boulogne-Billancourt, France) according to manufacturer’s recommendations, adapted to an ETI-MAX®3000 analyzer (DiaSorin, Gerenzano, Italy). Briefly, 50 µL per well of plasma or standards consisting of recombinant SARS–CoV-2 N-Antigenemia were deposited in a 96-well microplate coated with anti-SARS–CoV-2 N-Antigenemia antibodies; 50 µL of a solution containing a biotinylated–anti-SARS–CoV-2 N-Antigenemia antibodies were added and incubated at 37°C for 60 minutes; plates were washed with a phosphate buffer solution, and 100 µL of a solution containing horseradish peroxidase-conjugated streptavidin were added, followed by incubation for 30 minutes at 37°C; plates were washed with the phosphate buffer solution; 50 µL of a solution containing the TMB (3,3′,5,5′-tetramethylbenzidine) substrate and 50 µL of a second solution containing urea were added; after 15 minutes at 37°C, the colorimetric reaction was stopped by adding 50 µL of sulfuric acid (H_2_SO_4_). Absorbance values were measured at 450 nm, with a reference set at 630 nm. The N-Antigenemia-positivity threshold was 2.97 pg/mL.

**SARS–CoV-2 Reverse Transcriptase–Polymerase Chain Reaction (RT-PCR)**

Viral RNA in plasma was detected with the Simplexa™ COVID-19 Direct assay (DiaSorin [DiaSorin SA, Antony, France]), targeting the viral *ORF1ab* and *S* (spike) genes, and including an RNA internal control to detect RT-PCR failure and/or inhibition. Briefly, 50 μL of sample and 50 μL of reaction mix were added to their specific wells on a direct amplification disk, which was loaded onto the LIAISON® MDX instrument (DiaSorin).

**Anti-Human Leucocyte-Antigen (HLA) Antibodies: Quantification and Analysis**

Anti-HLA antibodies were identified with Luminex single antigen beads (One Lambda, Canoga Park, CA) before UC-MSC infusion and on D14 in the HLA Immunology Laboratory, Hôpital Saint-Louis, Paris, France (cut-off mean fluorescence intensity (MFI) > 500) on D0 and D14. Sera were tested using LabScreen Single Antigen Beads (SAB; One Lambda). LabScreen assays were performed according to the manufacturer’s protocol. After sampling, blood was centrifuged (within 4 days), and immediately frozen at –30°C. Before SAB testing, a 0.1-M solution of disodium EDTA (Sigma-Aldrich, St Louis, MO), pH=7.4, was diluted 1:10 in the patient’s serum and incubated for 10 minutes to avoid a prozone effect. A preliminary adsorption with microparticles treated with blocking solution was done to reduce high background noise caused by nonspecific binding of materials in human serum to the latex beads used in flow-cytometry antibody-detection assays. 20 µL of test serum were pre-added to 5 µL each of LS1A04 or LS2A01 SA beads, incubated in the dark for 30 minutes at room temperature, and then washed with wash buffer. 100 µL of goat anti-human IgG secondary antibody conjugated with R-phycoerythrin (PE), diluted 1:100, were added to the beads, incubated for 30 minutes in the dark at room temperature, then washed and read on the LABScan 200 flow cytometer (One Lambda). Panel reactive antibodies were calculated (screened) with the unacceptable (background) antigens (MFI > 1000) using the Eurotransplant Reference Laboratory (ETRL) virtual PRA calculator (<https://www.etrl.org/vPRA.aspx>). A PRAc differing from 0% on D0, identified pre-formed donor-specific antibodies (DSA) directed against the administered UC-MSCs in the absence of immunising events (pregnancy, transfusion, organ transplantation). De novo DSA were tracked on D14 based on increased anti-HLA immunisation with the presence of DSA directed against the administered UC-MSCs.

**Statistical Analyses**

Secondary analyses were considered exploratory. Time to death was compared between groups using the log-rank test. In other time-to-event analyses, death was considered as a competing event using the Fine & Gray approach.

**Role of the Funding Source**

The funder of the study had no role in the study design, data collection, data analysis, data interpretation, or writing of this report. The corresponding author had full access to all the study data and had final responsibility for the decision to submit for publication.

**RESULTS**

**Characterization of UC-MSC–based Advanced Therapy Medicinal Product (ATMP)**

Safety assays satisfied pre-set specifications for all batches, with the absence of microbiological organisms, endotoxins (<2 EU/mL), and mycoplasma (<10 CFU/mL). Before cell cryopreservation, each batch had a normal karyotype (Additional Table 1). Characterization was consistent across batches (Additional Table 2 and 3).

**Plasma inflammatory cytokines, chemokines, growth Factors and biomarkers at baseline (D0) and D2, D4, D7, and D14 after starting infusions.**

After Benjamini–Hochberg correction, biomarker concentrations did not differ between groups at any time evaluated (Additional Figure 2A and 3). Over time, several cytokines and chemokines (RAGE, MCP-2, IP-10, IL-7, IL-10, IL-13, IL-18 TNF-α) in UC-MSC–group patients decreased significantly, but those diminutions appeared later (D7 and D14 *vs* D0) (Additional Figure 2B and 3) than the placebo group’s pro-inflammatory cytokine and chemokine (RAGE, IP-10, MCP-3, MCP-1, MCP-2, CXCL9, M-CSF, IL-7, IL-5, IL-18, IL-27, CTACK) concentrations, which declined significantly on D4 and D7 *vs* D0 (Additional Figure 2C and 3).

**SARS–CoV-2 RT-PCR and SARS-CoV-2 N-Antigenemia Assay**

Based on viral RNA levels, 13 (28∙9%) patients had detectable viremia on D0 (Additional Figure 4A), while 42 (93∙3%) patients were N-antigenemia–positive (Additional Figure 4B). The percentages of viremic patients and N-antigenemia levels decreased sharply until D4 (Additional Figure 4A–C). No between-group differences were observed for the percentages of viremic patients and/or N-antigenemia diminutions over time up to D14.

**References**

1. Nicotra T, Desnos A, Halimi J, et al. Mesenchymal stem/stromal cell quality control: validation of mixed lymphocyte reaction assay using flow cytometry according to ICH Q2(R1). *Stem Cell Res Ther* 2020;11:426.

**Figure legend**

**Additional Figure 1. Survival probabilities and SOFA scores. (**A) Survival rates from D0 to D28 were comparable for the two groups (P=0∙63, log-rank test). (B) SOFA-score evolutions from D0 to D28 did not differ (P=0∙79, Wilcoxon test). Data are expressed as mean per day±standard deviation. *D* day. *SOFA* Sequential Organ-Failure Assessment score. *UC-MSCs* umbilical cord-derived mesenchymal stromal cells.

**Additional Figure 2. Analysis of plasma inflammatory cytokines, chemokines, growth factors, and biomarkers at baseline (D0) and D2, D4, D7, and D14 after starting infusions.** Concentrations of 48 cytokines were quantified in plasma from UC-MSC– (n=20) or placebo- treated (n =21) patients. Statistical analyses with Wilcoxon rank-sum tests compared values between groups (A) and within each group (B and C) at each day indicated. Volcano plots were generated for each comparison to show the log_2_ fold-changes relative to placebo or D0, with statistical significance reported as –log_10_ *P*-values. Significance, defined as *P*<0∙05, is in *blue,* with those remaining statistically significant after multiple corrections (Benjamini–Hochberg correction) in *red D* day, *D0* baseline, *UC-MSC* umbilical cord-derived mesenchymal stromal cell, *ANGP-1* angiopoietin-1, *BCA-1* B-cell–attracting chemokine-1, *CTACK* cutaneous T-cell–attracting chemokine, *CXCL-9* chemokine (C-X-C motif) ligand 9, *EGF* epidermal growth factor, *FLT-3L* Fms-related tyrosine kinase-3 ligand, *G-CSF* granulocyte-colony–stimulating factor, *IFN-γ* interferon-γ, *IL* interleukin, *IP-10* interferon gamma-induced protein-10, *MCP* monocyte chemoattractant protein, *M-CSF* macrophage-colony–stimulating factor, *MDC* macrophage-derived chemokine, *PDGF-AA* platelet-derived growth factor-AA, *RAGE* receptor for advanced glycation end products, *sCD40L* soluble cluster of differentiation-40 ligand, *SDF* stromal cell-derived factor, *SPD* surfactant protein B, *TGF-α* transforming growth factor-α, *TPO* thrombopoietin, *VEGF-A* vascular endothelial growth factor-A.

**Additional Figure 3. Analysis of plasma inflammatory cytokine, chemokine, growth factor, and biomarker concentrations on D0 (baseline), D2, D4, D7, and D14 after starting infusions.** The figure reports the quantification results for 10 cytokines selected among the 48 sought in plasma samples from patients treated with UC-MSCs (n=20) or placebo (n=21). Data are log_2_ transformed. Box plots of PaO_2_/FiO_2_ ratios: internal *horizontal lines* are the medians, *lower* and *upper box limits* are the 25^th^ and 75^th^ interquartile range, respectively, vertical bars are drawn down to the 10^th^ percentile and up to the 90^th^ percentile. *D* day, *IL* interleukin. *IP-10* interferon-gamma–induced protein-10, *MCP* monocyte chemoattractant protein, *RAGE* receptor for advanced glycation end products, *SDF-1* stromal cell-derived factor-α, *UC-MSCs* umbilical cord-derived mesenchymal stromal cells.

**Additional Figure 4. Analysis of plasma SARS–CoV-2 RNA and N-antigenemia levels at baseline (D0) and D2, D4, D7, and D14 after starting infusions.** Plasma SARS–CoV-2 RNA (by RT-PCR) and N-antigenemia in UC-MSC– (n=21, red) or placebo-treated (n=24, blue) patients were quantified. Based on viral RNA levels (A), five (23∙8%) UC-MSC– and eight (33∙3%) placebo-treated patients had detectable viremia on D0, while (B) N-antigenemia was positive for 20 (95∙2%) and 22 (91∙7%) patients, respectively. (C) Plasma SARS–CoV-2 NAg-level change from D0 to D14. Data are expressed as mean±standard deviation. The percentage of viremic patients and N-antigenemia levels decreased sharply until D4 (A–C). No between-group difference was observed in terms of percentage of viremic patients and/or decline from D0 to D14. Red=UC-MSC group; blue=placebo group. *D* day, *PaO_2_/FiO_2_* ratio of partial pressure of oxygen to fractional inspired oxygen, *RT-PCR* reverse transcription-polymerase chain reaction, *SARS–CoV-2* severe acute respiratory syndrome coronavirus-2, *UC-MSCs* umbilical cord-derived mesenchymal stromal cells.

***Additional Table 1:* Quality-control characteristics of UC-MSCs used as treatment: identity and safety.**

|  | **Results** | **Specifications** | **Compliance rate** |
| --- | --- | --- | --- |
| Identity |  |  |  |
| UC-MSC×10^6^/kg | 0∙9±0∙1 | 1∙0±0∙1* | 96% |
| Viability, % | 78∙4±5∙3 | ≥70 | 100% |
| CD90, % | 99∙2±1∙6 | >90 | 100% |
| CD73, % | 99∙9±0∙1 | >90 | 100% |
| CD105, % | 97∙0±1∙9 | >90 | 100% |
| CD45, CD34, CD11b, CD19, HLA-DR, % | 0∙8±0∙7 | <2 | 100% |
| Safety |  |  |  |
| Karyotype | Normal | Normal | 100% |
| Microbiology | Negative | Negative | 100% |
| Endotoxins | <2 EU/mL | <2 EU/mL | 100% |
| Mycoplasma | <10 CFU/mL | <10 CFU/mL | 100% |

Values are expressed as mean±standard deviation. *1.00±0.1×10^6^/kg with a maximum total dose of 80×10^6^ UC-MSCs.

*CD* cluster of differentiation, *EU* endotoxin unit, *CFU* colony-forming unit, *UC-MSCs* umbilical cord-derived mesenchymal stromal cells.

***Additional Table 2:* Quality-control characteristics of batch-1 UC-MSCs used as treatment: identity and safety.**

| **Criteria** | **Methods** | **Specifications** | **Results** | | |
| --- | --- | --- | --- | --- | --- |
| Viability | NC-200™ | ≥80% | 95% | 89% | 86% |
| Immunophenotype | Flow cytometry (BD Stemflow hMSC Analysis Kit) | Positive markers : CD90, CD73, CD105 ≥ 90%  Negative markers : CD45, CD34, CD11b, CD19, HLA-DR ≤ 2% | CD90 : 99.96%,  CD73 : 99.97%  CD105 : 97.86%  Negativ markers : 0.38% | CD90 : 99.67%,  CD73 : 99.97%  CD105 : 98.75%  Negativ markers : 1.59% | CD90 : 99.85%,  CD73 : 99.96%  CD105 : 98.37%  Negativ markers : 1.69% |
| karyotype | G-banding | Normal over at least 20 mitosis | Conform | Conform | Conform |
| Sterility | BactAlert | Negative | Negative | Negative | Negative |
| Mycoplasma | qPCR (Kit Venor Gem qEP Minerva) | < 10 CFU/mL | < 10 CFU/mL | < 10 CFU/mL | < 10 CFU/mL |
| Endotoxins | Kinetic colorimetry | < 2 EU/mL | < 2 EU/mL | < 2 EU/mL | < 2 EU/mL |
| CFU-F | CFU-F assay | ≥ 10/1000 nucleated cells | ≥ 10/1000 nucleated cells | ≥ 10/1000 nucleated cells | ≥ 10/1000 nucleated cells |

*CD* cluster of differentiation, *CFU* colony-forming unit, *CFU-F* colony-forming unit–fibroblast, *EU* endotoxin unit, *qPCR* quantitative polymerase chain reaction, *UC-MSCs* umbilical cord-derived mesenchymal stromal cells.

***Additional Table 3:* Quality-control characteristics of batch-2 UC-MSCs used as treatment: identity and safety.**

| **Criteria** | **Methods** | **Specifications** | **Results** | | |
| --- | --- | --- | --- | --- | --- |
| Viability | NC-200™ | ≥80% | 88% | 91% | 92% |
| Immunophenotype | Flow cytometry (BD Stemflow hMSC Analysis Kit) | Positive markers : CD90, CD73, CD105 ≥ 90%  Negative markers : CD45, CD34, CD11b, CD19, HLA-DR ≤ 2% | CD90: 99,77%  CD73: 99,94%  CD105: 99,24%  CD45 / HLA-DR / CD19 / CD14 : 0,31% | CD90: 99,97%  CD73: 99,89%  CD105: 99,47%  CD45 / HLA-DR / CD19 / CD14 : 1,28 | CD90: 99,53%  CD73: 99,86%  CD105: 96,53%  CD45 / HLA-DR / CD19 / CD14 : 1,23% |
| karyotype | G-banding | Normal over at least 20 mitosis | Conform | Conform | Conform |
| Sterility | BactAlert | Negative | Negative | Negative | Negative |
| Mycoplasma | qPCR (Kit Venor Gem qEP Minerva) | < 10 CFU/mL | < 10 CFU/mL | < 10 CFU/mL | < 10 CFU/mL |
| Endotoxins | Kinetic colorimetry | < 2 EU/mL | < 2 EU/mL | < 2 EU/mL | < 2 EU/mL |
| MLR | Evaluation of the lymphocyte proliferation inhibition by MSCs (flow cytometry) * | AUC* | 0.1522 | 0.1381 | 0.1582 |
| CFU-F | CFU-F assay | ≥ 10/1000 nucleated cells | ≥ 10/1000 nucleated cells | ≥ 10/1000 nucleated cells | ≥ 10/1000 nucleated cells |

*No published specifications.

*AUC* area under curve*, CD* cluster of differentiation, *CFU* colony-forming unit, *CFU-F* colony-forming unit–fibroblast, *EU* endotoxin unit, *qPCR* quantitative polymerase chain reaction, *UC-MSCs* umbilical cord-derived mesenchymal stromal cells.

***Additional Table 4:* Evolution of respiratory characteristics from baseline (D0) to D28**

|  | **UC-MSC (n=21)** | | | | | **Placebo (n=24)** | | | | | |
| --- | --- | --- | --- | --- | --- | --- | --- | --- | --- | --- | --- |
|  | D0 | D3 | D7 | D14 | D28 | D0 | D3 | D7 | D14 | D28 |  |
| Ventilatory support* | 10 (47∙6%) | 9 (42∙9%) | 4 (19∙0%) | 1 (4∙8%) | 0 (0%) | 4 (16∙7%) | 4 (16∙7%) | 2 (8∙3%) | 2 (8∙3%) | 0 (0%) |  |
| Invasive mechanical ventilation | 11  (52∙4%) | 12  (57∙1%) | 12/20 (60%) | 7/16  (43∙8%) | 4/4 (100%) | 20  (83∙3%) | 19  (79∙2%) | 16/22 (72∙7%) | 10/16 (62∙5%) | 5/6 (83∙3%) |  |
| Tidal volume, mL/kg | 6∙2 (0∙7) | 7∙0 (1∙4) | 7 (1∙2) | 6∙0 (0∙9) | 5∙7 (0∙8) | 6∙3 (0∙8) | 6∙8 (1∙4) | 7∙1 (2∙2) | 7∙0 (1∙5) | 6∙7 (1∙7) |  |
| Plateau airway pressure, cm H_2_O | 21∙8 (4∙2) | 22∙2 (5∙4) | 24∙8 (7∙0) | 26∙0 (5∙7) | 45∙5 (2∙1) | 24∙8 (5∙0) | 26∙4 (8∙3) | 26∙5 (2∙4) | 24∙7 (8∙6) | 18 (NA) |  |
| PEEP | 10∙8 (2∙9) | 10∙7 (3∙7) | 10∙4 (3∙9) | 10∙3 (4∙6) | 6∙2 (1∙9) | 11∙2 (3∙2) | 9∙4 (3∙2) | 8∙9 (3∙7) | 9∙7 (4∙2) | 7∙8 (4∙2) |  |
| Driving pressure | 11∙3 (4∙3) | 11∙4 (4∙0) | 15∙2 (6∙3) | 16 (5∙7) | 38∙5 (4∙9) | 13∙2 (3∙9) | 16∙2 (8∙9) | 15∙5 (2∙6) | 14∙0 (6∙1) | 13∙0 (NA) |  |
| Compliance, mL/cm H_2_O | 45∙2 (27∙8) | 42∙9 (12∙8) | 37∙6 (19∙8) | 26∙6 (9∙5) | 11∙1 (2∙3) | 35∙2 (14∙9) | 34∙6 (12∙2) | 27∙7 (4∙9) | 44∙8 (40∙0) | 52∙1 (NA) |  |
| Neuromuscular blocking agents | 6  (28∙6%) | 9  (42∙9%) | 6/19 (31∙6%) | 3/14 (21∙4%) | 1/4  (25%) | 16  (66∙7%) | 14  (58∙3%) | 7/20  (35%) | 5/15 (33∙3%) | 1/6  (16∙7%) |  |
| Ventilation mode |  |  |  |  |  |  |  |  |  |  |  |
| Volume control | 11/11  (100%) | 9/12  (75%) | 8/12 (66∙7%) | 5/7  (71∙4%) | 2/4  (50%) | 19/20  (95%) | 18/19  (94∙7%) | 13/16  (81∙3) | 8/10  (80%) | 2/5  (40%) |  |
| Pressure control | 0 (0%) | 0 (0%) | 0 (0%) | 0 (0%) | 0 (0%) | 0 (0%) | 0 (0%) | 0 (0%) | 0 (0%) | 0 (0%) |  |
| Pressure support | 0 (0%) | 3 (25%) | 4 (33∙3) | 2 (28∙6%) | 2 (50%) | 1 (5%) | 1 (5∙2%) | 3 (18∙7%) | 2 (20%) | 3 (60%) |  |

Values are expressed mean (standard or number) (%). Denominators are indicated when values were missing. *D* day, *PaO_2_/FiO_2_* ratio of partial pressure of oxygen to fractional inspired oxygen, *PEEP* positive end-expiratory pressure, *UC-MSCs* umbilical cord-derived mesenchymal stromal cells, *NA* not applicable.

*Non-invasive ventilation and/or high-flow nasal oxygen therapy

***Additional Table 5.* UC-MSCs posology per patient**

| **Patient** | **Weight (kg)** | **Dose ×10^6^ UC-MSCs/kg (D1, D3±1, and D5±1)** |
| --- | --- | --- |
| R0003 | 100∙0* | 0∙8 D1, D3, and D4 |
| R0004 | 89∙0* | 0∙9 D1, D3, and D4 |
| R0008 | 75∙0 | 1∙0 D1, D4, and D6 |
| R0123 | 91∙0* | 0∙9 D1, D3, and D5 |
| R0181 | 80∙0 | 1∙0 D1, and D3 |
| S2009 | 94∙0* | 0∙9 D1, D2, and D4 |
| S2114 | 85∙0* | 0∙9 D1 |
| S2115 | 85∙0* | 0∙9 D1, D4, and D6 |
| S2118 | 92∙0* | 0∙9 D1, and D4 |
| S2119 | 70∙0 | 1∙0 D1, D3, and D4 |
| S2121 | 85∙0* | 0∙9 D1, D2, and D6 |
| S2124 | 127∙0* | 0∙6 D1, D3, and D4 |
| S2125 | 87∙0* | 0∙9 D1, D3, and D6 |
| S2128 | 80∙0 | 1∙0 D1, D4, and D6 |
| S2129 | 92∙0* | 0∙7 D1^†^, 0∙8 D3, 0∙9 D4 |
| S2132 | 81∙0* | 1∙0 D1, 1∙0 D3, 1∙0 D4 |
| S2134 | 75∙0 | 1∙0 D1, 1∙0 D3, 0∙9 D4^†^ |
| S2189 | 87∙0* | 0∙9 D1, D3, and D4 |
| S2192 | 80∙0 | 1∙0 D1 |
| S2194 | 85∙0* | 0∙9 D1, D3, and D4 |
| S2196 | 75∙0 | 1∙0 D1, 1∙0 D3, 1∙0 D6 |

*Patients for whom 1∙0±0∙1×10^6^/kg reached a maximum total dose of 80×10^6^ MSC.

^†^Doses below the pre-specified therapeutic dose (1∙0±0∙1×10^6^/kg, with a maximum total dose of 80×10^6^ MSC). *D* day, *UC-MSCs* umbilical cord-derived mesenchymal stromal cells.

***Additional Table 6. Subgroup analyses of the primary outcome.***

| **Imputation*** | **Sub group** | **UC-MSC**  **(n = 21)** | **placebo**  **(n = 24)** | **Estimate** | **p** |
| --- | --- | --- | --- | --- | --- |
| 1 | MV = 0 | 58.7 [33.4;105.3] | 95.2 [61.4;125.4] | 0.4 [-133.6;134.4] | 0.99 |
| 2 | MV = 0 | 58.7 [33.4;101.9] | 83.7 [61.4;101.7] | 5.1 [-113.8;123.9] | 0.93 |
| 1 | MV = 1 | 54.3 [-45.7;78.3] | 6.9 [-41.8;79.7] | 0.6 [-64.6;65.7] | 0.99 |
| 2 | MV = 1 | 54.3 [-36.5;78.3] | 6.9 [-41.8;69.8] | 10.8 [-46.8;68.4] | 0.70 |
| 1 | PaO_2_/FiO_2_ ratio > 150 | -12.7 [-76.2;70.8] | 71.7 [-52.6;110] | -4.6 [-105.6;96.4] | 0.93 |
| 2 | PaO_2_/FiO_2_ ratio > 150 | -12.7 [-62.1;70.8] | 69.2 [-52.6;97.6] | -4.8 [-94.8;85.1] | 0.91 |
| 1 | PaO_2_/FiO_2_ ratio ≤ 150 | 63.0 [54.3;100.6] | 17.9 [-7.2;53.7] | 39.2 [-8.4;86.8] | 0.10 |
| 2 | PaO_2_/FiO_2_ ratio ≤ 150 | 63.0 [54.3;100.6] | 17.9 [-7.2;53.7] | 41.5 [-1.2;84.3] | 0.06 |
| 1 | Age < 65 years | 73.8 [47.9;109.9] | 77.1 [-2.5;112.3] | 27.9 [-63.9;119.8] | 0.53 |
| 2 | Age < 65 years | 73.8 [47.9;109.9] | 70.4 [-2.5;85.8] | 31.3 [-49.4;112.1] | 0.42 |
| 1 | Age ≥ 65 years | 46.7 [-51.8;63.3] | -18.6 [-60.8;37.9] | 10.6 [-59.6;80.9] | 0.76 |
| 2 | Age ≥ 65 years | 46.7 [-51.8;63.3] | -18.6 [-60.8;37.9] | 20.0 [-42.0;82.0] | 0.51 |
| 1 | DSA negative among treated group | 58.8 [-42.7;107.3] | 25.3 [-33.3;104.6] | 13.9 [-48.3;76.1] | 0.65 |
| 2 | DSA negative among treated group | 58.8 [-42.7;107.3] | 25.3 [-33.3 ;83.1] | 18.4 [-36.1;72.9] | 0.50 |

Values are median [interquartile range]. *MV* mechanical ventilation, *DSA* donor-specific antigen, *UC-MSCs* umbilical cord-derived mesenchymal stromal cells.

**Imputation 1*: PaO2/FiO2 ratios of patients who died were imputed to 50, whereas those who improved and left the ICU before D7 were imputed using their last observed value plus 10%.

**Imputation 2:* A sensitivity analysis was computed using a last observed carried forward approach.

***Additional Table 7. Complete list of adverse events.***

| **Adverse events** | **UC-MSCs** | **Placebo** | **Total** |
| --- | --- | --- | --- |
| Edema | 0 (0%) | 1 (1.4%) | 1 (0.7%) |
| Atrial Fibrillation | 1 (1.5%) | 0 (0%) | 1 (0.7%) |
| Metabolic acidosis | 1 (1.5%) | 0 (0%) | 1 (0.7%) |
| Worsening hypercapnia | 0 (0%) | 1 (1.4%) | 1 (0.7%) |
| Worsening hepatic cytolysis | 0 (0%) | 1 (1.4%) | 1 (0.7%) |
| Worsening shock | 0 (0%) | 1 (1.4%) | 1 (0.7%) |
| Worsening interstitial syndrome | 0 (0%) | 1 (1.4%) | 1 (0.7%) |
| Delirium | 2 (2.9%) | 1 (1.4%) | 3 (2.1%) |
| Metabolic alkalosis | 1 (1.5%) | 0 (0%) | 1 (0.7%) |
| Anemia | 1 (1.5%) | 0 (0%) | 1 (0.7%) |
| Anemia requiring transfusion | 0 (0%) | 1 (1.4%) | 1 (0.7%) |
| Anemia – Hb 7.8 g/dL | 1 (1.5%) | 0 (0%) | 1 (0.7%) |
| Microscopic hematuria | 1 (1.5%) | 0 (0%) | 1 (0.7%) |
| Cardiac arrest and left pneumothorax | 1 (1.5%) | 0 (0%) | 1 (0.7%) |
| Increase in triglycerid and lactatate-deshydrogenase levels | 0 (0%) | 1 (1.4%) | 1 (0.7%) |
| Pseudomonas aeruginosa bacteremia | 1 (1.5%) | 0 (0%) | 1 (0.7%) |
| Bronchospasm | 0 (0%) | 1 (1.4%) | 1 (0.7%) |
| Hypovolemic shock | 1 (1.5%) | 0 (0%) | 1 (0.7%) |
| Refractory septic shock with bacteremia | 0 (0%) | 1 (1.4%) | 1 (0.7%) |
| Septic shock related to haemophilus pneumonia | 0 (0%) | 1 (1.4%) | 1 (0.7%) |
| Refractory septic shock associated with multiple organ failure | 1 (1.5%) | 0 (0%) | 1 (0.7%) |
| Cholestasis | 1 (1.5%) | 0 (0%) | 1 (0.7%) |
| Fall from standing position | 0 (0%) | 1 (1.4%) | 1 (0.7%) |
| Acute cor pulmonale | 1 (1.5%) | 0 (0%) | 1 (0.7%) |
| Increase in D-Dimer | 1 (1.5%) | 0 (0%) | 1 (0.7%) |
| Terminal extubation for irreversible encephalopathy | 1 (1.5%) | 0 (0%) | 1 (0.7%) |
| Psychiatric decompensation | 1 (1.5%) | 0 (0%) | 1 (0.7%) |
| Acute respiratory distress requiring intubation and mechanical ventilation | 1 (1.5%) | 0 (0%) | 1 (0.7%) |
| Shock | 1 (1.5%) | 4 (5.6%) | 5 (3.6%) |
| Multiple organ failure | 1 (1.5%) | 0 (0%) | 1 (0.7%) |
| Refractory multiple organ failure | 0 (0%) | 1 (1.4%) | 1 (0.7%) |
| Acute renal failure - anuria | 0 (0%) | 1 (1.4%) | 1 (0.7%) |
| Worsening renal failure | 1 (1.5%) | 0 (0%) | 1 (0.7%) |
| Worsening shock | 0 (0%) | 1 (1.4%) | 1 (0.7%) |
| Worsening shock and respiratory distress | 0 (0%) | 1 (1.4%) | 1 (0.7%) |
| Severe worsening shock and respiratory distress | 1 (1.5%) | 0 (0%) | 1 (0.7%) |
| Worsening respiratory status with fever | 1 (1.5%) | 0 (0%) | 1 (0.7%) |
| Worsening respiratory distress requiring intubation and mechanical ventilation | 1 (1.5%) | 0 (0%) | 1 (0.7%) |
| Worsening hemodynamic status | 0 (0%) | 1 (1.4%) | 1 (0.7%) |
| Delirium and neuromyopathy | 1 (1.5%) | 0 (0%) | 1 (0.7%) |
| Mixt delirium | 0 (0%) | 1 (1.4%) | 1 (0.7%) |
| Respiratory exhaustion - hypoxemia | 1 (1.5%) | 0 (0%) | 1 (0.7%) |
| Dialysis | 0 (0%) | 1 (1.4%) | 1 (0.7%) |
| Diarhhea | 0 (0%) | 1 (1.4%) | 1 (0.7%) |
| Liquid diarrhea | 1 (1.5%) | 0 (0%) | 1 (0.7%) |
| Increase in uremia | 0 (0%) | 1 (1.4%) | 1 (0.7%) |
| Acute pulmonary embolism | 0 (0%) | 1 (1.4%) | 1 (0.7%) |
| *Pseudomonas aeruginosa*- related ventilator-associated pneumonia | 1 (1.5%) | 0 (0%) | 1 (0.7%) |
| Thrombopenia | 0 (0%) | 1 (1.4%) | 1 (0.7%) |
| Isolated thrombopenia | 0 (0%) | 1 (1.4%) | 1 (0.7%) |
| Right occipital eschar | 0 (0%) | 1 (1.4%) | 1 (0.7%) |
| Labbial eschar | 1 (1.5%) | 0 (0%) | 1 (0.7%) |
| Heel, seat, elbow pressure sores | 1 (1.5%) | 0 (0%) | 1 (0.7%) |
| Occipital eschar stade 4 | 0 (0%) | 1 (1.4%) | 1 (0.7%) |
| Fever | 0 (0%) | 1 (1.4%) | 1 (0.7%) |
| Fever 39.1°C | 1 (1.5%) | 0 (0%) | 1 (0.7%) |
| Fever 39.5°C | 1 (1.5%) | 0 (0%) | 1 (0.7%) |
| *Staphylococcus epidermidis* bacteremia | 0 (0%) | 1 (1.4%) | 1 (0.7%) |
| *Staphylococcus haemolyticus* bacteremia | 0 (0%) | 1 (1.4%) | 1 (0.7%) |
| *Streptococcus mitis* bacteremia | 1 (1.5%) | 0 (0%) | 1 (0.7%) |
| Hyperkalemia without renal failure | 1 (1.5%) | 0 (0%) | 1 (0.7%) |
| Persistent hyperkalemia related to acidosis | 0 (0%) | 1 (1.4%) | 1 (0.7%) |
| Hyperleukocytosis | 0 (0%) | 1 (1.4%) | 1 (0.7%) |
| Hyperlipasemia | 1 (1.5%) | 0 (0%) | 1 (0.7%) |
| Arterial hypertension | 0 (0%) | 1 (1.4%) | 1 (0.7%) |
| Hypertension | 1 (1.5%) | 0 (0%) | 1 (0.7%) |
| Hypoalbumonemia | 1 (1.5%) | 0 (0%) | 1 (0.7%) |
| Hypotension | 1 (1.5%) | 0 (0%) | 1 (0.7%) |
| Severe edema | 0 (0%) | 1 (1.4%) | 1 (0.7%) |
| Infection related to Pseudomonas aeruginosa and Klebsiella | 0 (0%) | 1 (1.4%) | 1 (0.7%) |
| Oropharyngeal infection | 1 (1.5%) | 0 (0%) | 1 (0.7%) |
| Septic shock with respiratory failure | 1 (1.5%) | 0 (0%) | 1 (0.7%) |
| Acute Renal Failure | 1 (1.5%) | 1 (1.4%) | 2 (1.4%) |
| Acute kidney injury | 0 (0%) | 1 (1.4%) | 1 (0.7%) |
| Renal Failure | 0 (0%) | 1 (1.4%) | 1 (0.7%) |
| Functional acute kidney injury | 1 (1.5%) | 0 (0%) | 1 (0.7%) |
| Stable acute renal failure | 0 (0%) | 1 (1.4%) | 1 (0.7%) |
| Mild hyponatremia | 1 (1.5%) | 0 (0%) | 1 (0.7%) |
| Skin lesions related to prone positioning | 0 (0%) | 1 (1.4%) | 1 (0.7%) |
| Lymphangitis | 0 (0%) | 1 (1.4%) | 1 (0.7%) |
| Right arm lymhangitis | 0 (0%) | 1 (1.4%) | 1 (0.7%) |
| Nausea and diarrhea | 1 (1.5%) | 0 (0%) | 1 (0.7%) |
| Severe neuromyopathy | 0 (0%) | 1 (1.4%) | 1 (0.7%) |
| Very severe neuromyopathy | 1 (1.5%) | 0 (0%) | 1 (0.7%) |
| Intensive care unit-associate neuromyopathy | 0 (0%) | 1 (1.4%) | 1 (0.7%) |
| Moderate neuromyopathy | 1 (1.5%) | 0 (0%) | 1 (0.7%) |
| New onset of *Pseudomonas aeruginosa*-related VAP | 0 (0%) | 1 (1.4%) | 1 (0.7%) |
| Post-extubation laryngeal edema | 0 (0%) | 1 (1.4%) | 1 (0.7%) |
| Alveolo-interstitiel syndrome | 0 (0%) | 1 (1.4%) | 1 (0.7%) |
| Pansinusitis requiring surgery | 1 (1.5%) | 0 (0%) | 1 (0.7%) |
| Right *Pseudomonas aeruginosa*-related VAP | 0 (0%) | 1 (1.4%) | 1 (0.7%) |
| *Pseudomonas aeruginosa-* and *Klebsiella-*related VAP | 1 (1.5%) | 0 (0%) | 1 (0.7%) |
| *Staphylococcus aureus-* and *Haemophilus-*related VAP | 1 (1.5%) | 0 (0%) | 1 (0.7%) |
| *Staphylococcus aureus*-related VAP | 0 (0%) | 1 (1.4%) | 1 (0.7%) |
| *Citrobacter-* and *Serratia-*related VAP | 0 (0%) | 1 (1.4%) | 1 (0.7%) |
| Methicillin-sensitive *Staphylococcus aureus-*related VAP | 0 (0%) | 1 (1.4%) | 1 (0.7%) |
| Late *Klebsiella-*related ventilator-associated pneumonia | 0 (0%) | 1 (1.4%) | 1 (0.7%) |
| Febrile peak | 0 (0%) | 1 (1.4%) | 1 (0.7%) |
| Fever 38.2°C | 0 (0%) | 1 (1.4%) | 1 (0.7%) |
| Hypertensive peak | 1 (1.5%) | 0 (0%) | 1 (0.7%) |
| VAP related to oropharyngeal flora | 0 (0%) | 1 (1.4%) | 1 (0.7%) |
| VAP | 1 (1.5%) | 0 (0%) | 1 (0.7%) |
| Late ventilator-associated pneumonia related to Citrobacter | 1 (1.5%) | 0 (0%) | 1 (0.7%) |
| VAP related to oropharyngeal flora | 0 (0%) | 1 (1.4%) | 1 (0.7%) |
| Healthcare-associated pneumonia | 0 (0%) | 1 (1.4%) | 1 (0.7%) |
| Pneumonia related to oropharyngeal flora | 0 (0%) | 1 (1.4%) | 1 (0.7%) |
| VAP related to multidrug-resistant bacteria | 1 (1.5%) | 0 (0%) | 1 (0.7%) |
| VAP related to oropharyngeal flora | 0 (0%) | 1 (1.4%) | 1 (0.7%) |
| VAP related to Hafnia and Proteus | 0 (0%) | 1 (1.4%) | 1 (0.7%) |
| VAP related to Klebsiella | 1 (1.5%) | 0 (0%) | 1 (0.7%) |
| VAP related to Pseudomonas spp. | 1 (1.5%) | 0 (0%) | 1 (0.7%) |
| Extracorporeal membrane oxygenator implantation | 1 (1.5%) | 0 (0%) | 1 (0.7%) |
| Refractory acute respiratory distress syndrome | 0 (0%) | 1 (1.4%) | 1 (0.7%) |
| Bacteremia related to cocci Gram+ bacteria | 1 (1.5%) | 0 (0%) | 1 (0.7%) |
| Refractory acute respiratory distress syndrome and multiple organ failure | 0 (0%) | 1 (1.4%) | 1 (0.7%) |
| Superinfection related to Moraxella | 1 (1.5%) | 0 (0%) | 1 (0.7%) |
| Suspected pulmonary embolism | 0 (0%) | 1 (1.4%) | 1 (0.7%) |
| Suspected propofol-related infusion syndrome | 0 (0%) | 1 (1.4%) | 1 (0.7%) |
| Suspected S.aureus-associated endocarditis | 1 (1.5%) | 0 (0%) | 1 (0.7%) |
| Bilateral alveolo-interstitial syndrome | 1 (1.5%) | 0 (0%) | 1 (0.7%) |
| Biological inflammatory syndrome | 1 (1.5%) | 0 (0%) | 1 (0.7%) |
| Tachycardia | 1 (1.5%) | 2 (2.8%) | 3 (2.1%) |
| Effort-related tachycardia | 2 (2.9%) | 0 (0%) | 2 (1.4%) |
| Increase in D-Dimer | 1 (1.5%) | 0 (0%) | 1 (0.7%) |
| High level of D-Dimer | 1 (1.5%) | 0 (0%) | 1 (0.7%) |
| Thrombocytemia | 0 (0%) | 1 (1.4%) | 1 (0.7%) |
| Thrombopenia | 0 (0%) | 1 (1.4%) | 1 (0.7%) |
| Tracheostomy for mechanical ventilation weaning | 1 (1.5%) | 0 (0%) | 1 (0.7%) |
| Vomitting | 1 (1.5%) | 0 (0%) | 1 (0.7%) |
| Total | 68 (48.9%) | 71 (51.1%) | 139 (100%) |

Values are expressed as number (%). *Hb* hemoglobin*, VAP* ventilator-associated pneumonia*.*

***Additional Table 8*. Summary of pre-specified infusion-associated adverse events for randomized subjects**

|  | **UC-MSC (n=21)** | **Placebo (n=24)** | **Total** |
| --- | --- | --- | --- |
| Pre-specified AEs occurring during treatment infusion |  |  |  |
| A clinical scenario consistent with transfusion incompatibility or  transfusion-related infection | 0 (0%) | 0 (0%) | 0 (0%) |
| Reaction necessitating treatment stoppage | 0 (0%) | 0 (0%) | 0 (0%) |
| Pre-specified AEs occurring within 6 h of treatment onset |  |  |  |
| Vasopressor-dose Increase | 0 (0%) | 1 (4.2%) | 1 (2.2%) |
| New ventricular tachycardia or fibrillation, or asystole | 0 (0%) | 1 (4.2%) | 1 (2.2%) |
| Worsening hypoxemia | 0 (0%) | 0 (0%) | 0 (0%) |
| Pre-specified AE occurring within 24 h of treatment infusion |  |  |  |
| Any cardiac arrest or death | 0 (0%) | 0 (0%) | 0 (0%) |

Values are expressed as number (%). *AE* adverse event, *UC-MSCs* umbilical cord-derived mesenchymal stromal cells.
