# Supplementary figures and images for: Treatment of COVID-19-associated ARDS with mesenchymal stromal cells: a multicenter randomized double-blind trial
Source: Crit Care. 2022 Feb 21;26:48. doi: 10.1186/s13054-022-03930-4 (PMC8860258; doi:10.1186/s13054-022-03930-4)

ADDITIONAL FIGURE 1

A

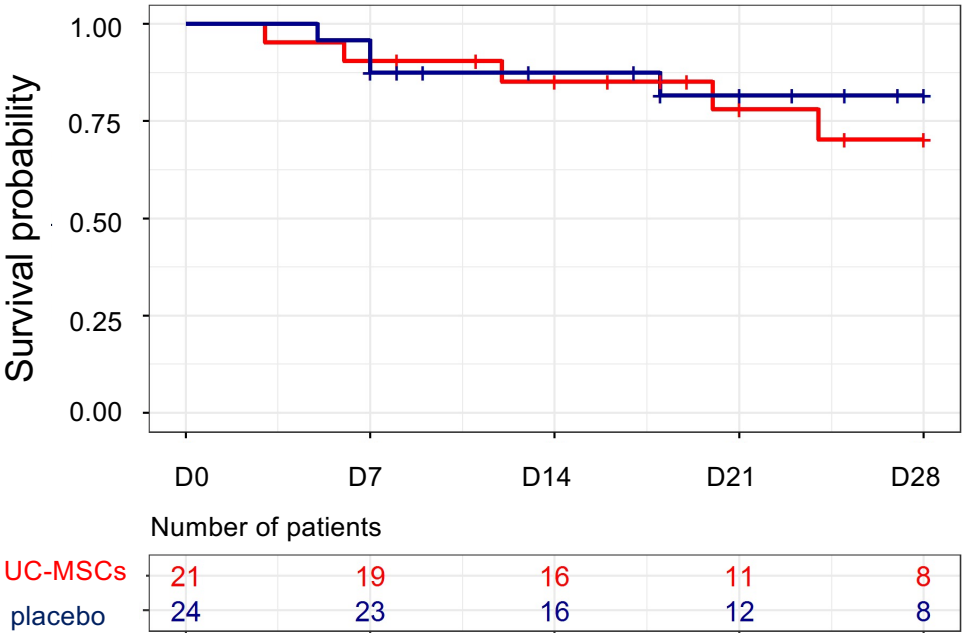

B

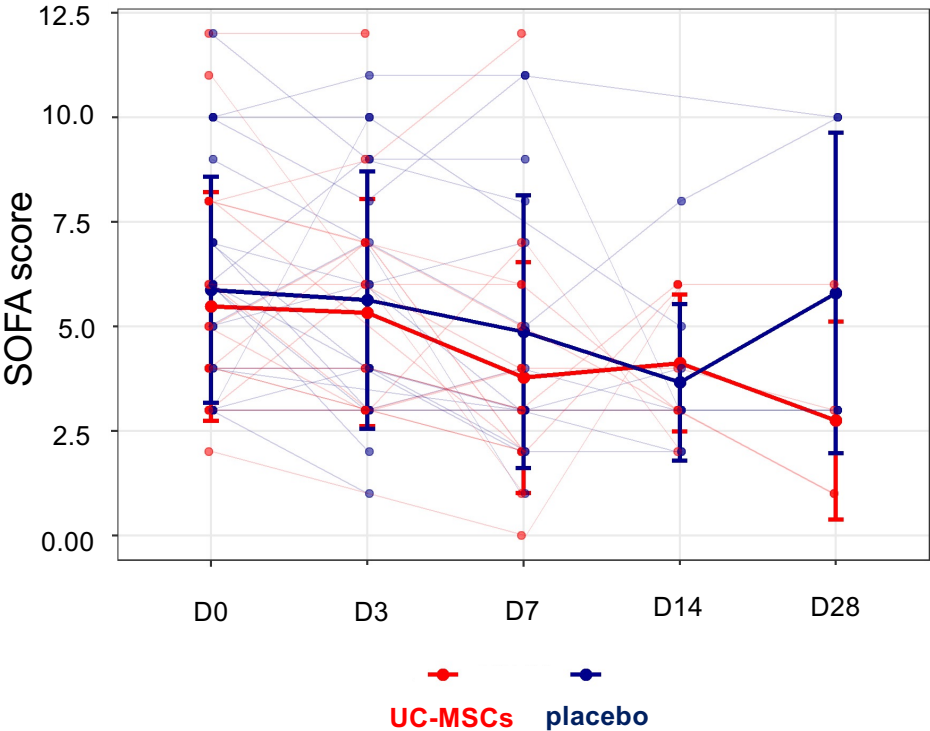

Supplement: Supplementary file 2 — Additional file 2: Figure S1. Survival probabilities and SOFA scores. (A) Survival rates from D0 to D28 were comparable for the two groups (P = 0.63, log-rank test). (B) SOFA-score evolutions from D0 to D28 did not differ (P = 0.79, Wilcoxon test). Data are expressed as mean per day ± standard deviation. D day. SOFA Sequential Organ-Failure Assessment score. UC-MSCs umbilical cord-derived mesenchymal stromal cells. [file 13054_2022_3930_MOESM2_ESM.pdf]

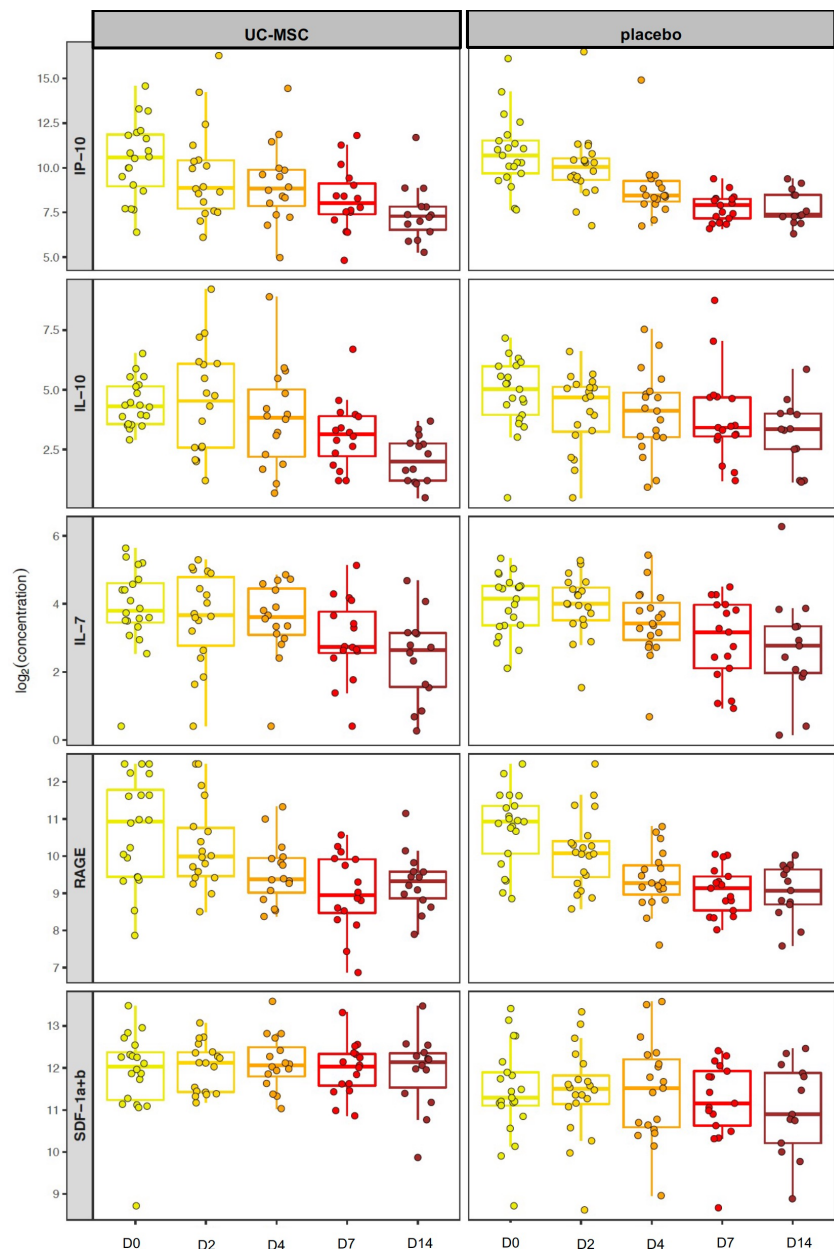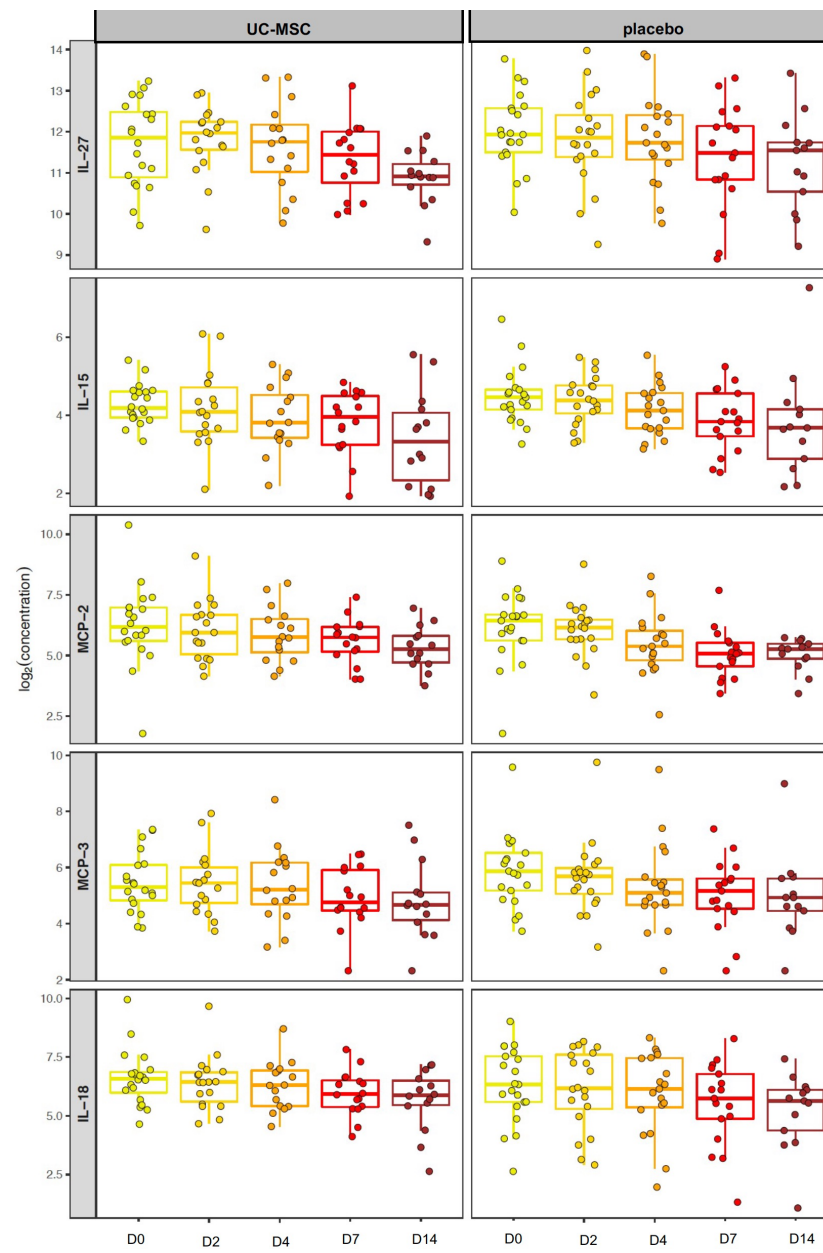

ADDITIONAL FIGURE 3

Supplement: Supplementary file 4 — Additional file 4: Figure S3. Analysis of plasma inflammatory cytokine, chemokine, growth factor, and biomarker concentrations on D0 (baseline), D2, D4, D7, and D14 after starting infusions. The figure reports the quantification results for 10 cytokines selected among the 48 sought in plasma samples from patients treated with UC-MSCs (n = 20) or placebo (n = 21). Data are log2 transformed. Box plots of PaO2/FiO2 ratios: internal horizontal lines are the medians, lower and upper box limits are the 25th and 75th interquartile range, respectively, vertical bars are drawn down to the 10th percentile and up to the 90th percentile. D day, IL interleukin. IP-10 interferon-gamma-induced protein-10, MCP monocyte chemoattractant protein, RAGE receptor for advanced glycation end products, SDF-1 stromal cell-derived factor-α, UC-MSCs umbilical cord-derived mesenchymal stromal cells. [file 13054_2022_3930_MOESM4_ESM.pdf]

ADDITIONAL FIGURE 4

A

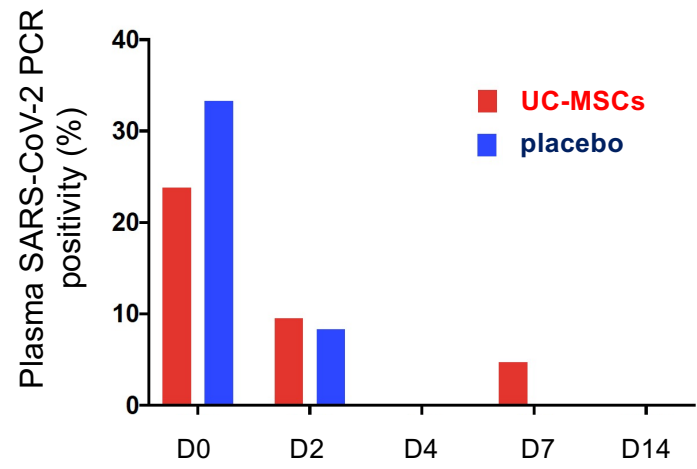

B

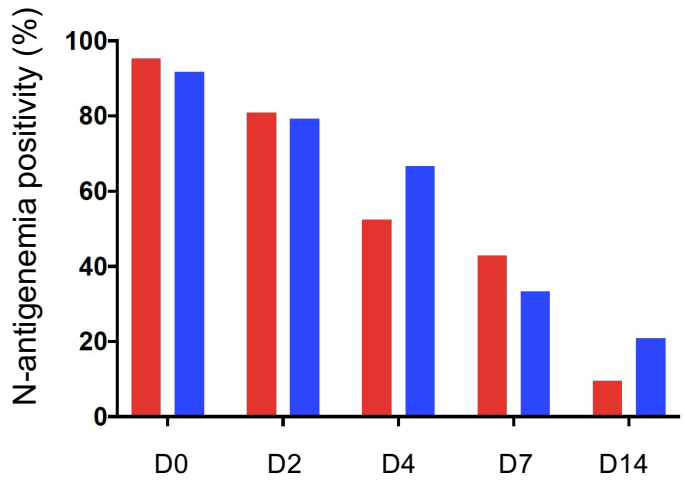

C

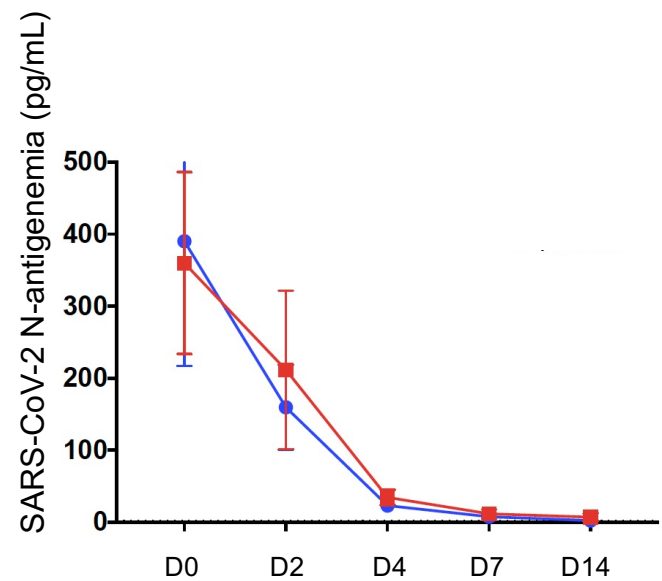

Supplement: Supplementary file 5 — Additional file 5: Figure S4. Analysis of plasma SARS–CoV-2 RNA and N-antigenemia levels at baseline (D0) and D2, D4, D7, and D14 after starting infusions. Plasma SARS–CoV-2 RNA (by RT-PCR) and N-antigenemia in UC-MSC– (n = 21, red) or placebo-treated (n = 24, blue) patients were quantified. Based on viral RNA levels (A), five (23.8%) UC-MSC– and eight (33.3%) placebo-treated patients had detectable viremia on D0, while (B) N-antigenemia was positive for 20 (95.2%) and 22 (91.7%) patients, respectively. (C) Plasma SARS–CoV-2 NAg-level change from D0 to D14. Data are expressed as mean ± standard deviation. The percentage of viremic patients and N-antigenemia levels decreased sharply until D4 (A–C). No between-group difference was observed in terms of percentage of viremic patients and/or decline from D0 to D14. Red = UC-MSC group; blue = placebo group. D day, PaO2/FiO2 ratio of partial pressure of oxygen to fractional inspired oxygen, RT-PCR reverse transcription-polymerase chain reaction, SARS–CoV-2 severe acute respiratory syndrome coronavirus-2, UC-MSCs umbilical cord-derived mesenchymal stromal cells. [file 13054_2022_3930_MOESM5_ESM.pdf]
