# Supplementary material for: Treatment of COVID-19-associated ARDS with mesenchymal stromal cells: a multicenter randomized double-blind trial
Source: Crit Care. 2022 Feb 21;26:48. doi: 10.1186/s13054-022-03930-4 (PMC8860258; doi:10.1186/s13054-022-03930-4)
Supplement: Supplementary file 3 — Additional file 3: Figure S2. Analysis of plasma inflammatory cytokines, chemokines, growth factors, and biomarkers at baseline (D0) and D2, D4, D7, and D14 after starting infusions. Concentrations of 48 cytokines were quantified in plasma from UC-MSC– (n = 20) or placebo- treated (n = 21) patients. Statistical analyses with Wilcoxon rank-sum tests compared values between groups (A) and within each group (B and C) at each day indicated. Volcano plots were generated for each comparison to show the log2 fold-changes relative to placebo or D0, with statistical significance reported as −log10 P-values. Significance, defined as P < 0.05, is in blue, with those remaining statistically significant after multiple corrections (Benjamini–Hochberg correction) in red D day, D0 baseline, UC-MSC umbilical cord-derived mesenchymal stromal cell, ANGP-1 angiopoietin-1, BCA-1 B-cell-attracting chemokine-1, CTACK cutaneous T-cell-attracting chemokine, CXCL-9 chemokine (C-X-C motif) ligand 9, EGF epidermal growth factor, FLT-3L Fms-related tyrosine kinase-3 ligand, G-CSF granulocyte-colony-stimulating factor, IFN-γ interferon-γ, IL interleukin, IP-10 interferon gamma-induced protein-10, MCP monocyte chemoattractant protein, M-CSF macrophage-colony-stimulating factor, MDC macrophage-derived chemokine, PDGF-AA platelet-derived growth factor-AA, RAGE receptor for advanced glycation end products, sCD40L soluble cluster of differentiation-40 ligand, SDF stromal cell-derived factor, SPD surfactant protein B, TGF-α transforming growth factor-α, TPO thrombopoietin, VEGF-A vascular endothelial growth factor-A. [file 13054_2022_3930_MOESM3_ESM.pdf]

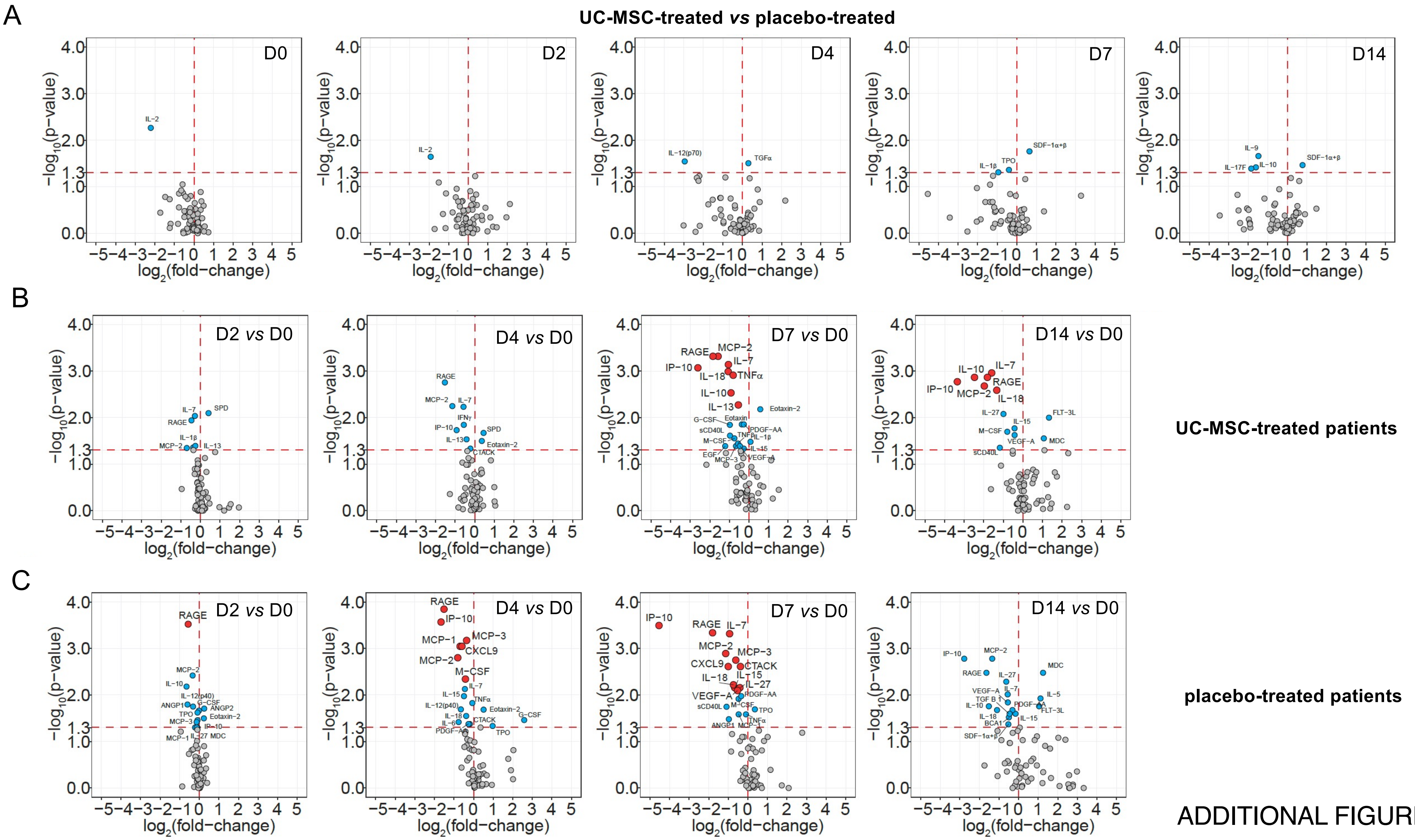

ADDITIONAL FIGURE 2
